# Statin-induced Mitochondrial Priming Sensitizes Multiple Myeloma Cells to BCL2 and MCL-1 Inhibitors

**DOI:** 10.1158/2767-9764.CRC-23-0350

**Published:** 2023-12-08

**Authors:** Dennis Juarez, Roberta Buono, Shannon M. Matulis, Vikas A. Gupta, Madeleine Duong, Jacob Yudiono, Madhuri Paul, Sharmila Mallya, Grace Diep, Peter Hsin, Alexander Lu, Sang Mi Suh, Vy M. Dong, Andrew W. Roberts, Joel D. Leverson, Muhammad Jalaluddin, Zhuangzhuang Liu, Orlando F. Bueno, Lawrence H. Boise, David A. Fruman

**Affiliations:** 1 Department of Molecular Biology and Biochemistry, University of California, Irvine, California.; 2 Department of Hematology and Medical Oncology and the Winship Cancer Institute at Emory University, Atlanta, Georgia.; 3 Department of Chemistry, University of California, Irvine, California.; 4 Oncology Development, AbbVie, Inc, North Chicago, Illinois.

## Abstract

The BCL2 inhibitor venetoclax promotes apoptosis in blood cancer cells and is approved for treatment of chronic lymphocytic leukemia and acute myeloid leukemia. However, multiple myeloma cells are frequently more dependent on MCL-1 for survival, conferring resistance to venetoclax. Here we report that mevalonate pathway inhibition with statins can overcome resistance to venetoclax in multiple myeloma cell lines and primary cells. In addition, statins sensitize to apoptosis induced by MCL-1 inhibitor, S63845. In retrospective analysis of venetoclax clinical studies in multiple myeloma, background statin use was associated with a significantly enhanced rate of stringent complete response and absence of progressive disease. Statins sensitize multiple myeloma cells to venetoclax by upregulating two proapoptotic proteins: PUMA via a p53-independent mechanism and NOXA via the integrated stress response. These findings provide rationale for prospective testing of statins with venetoclax regimens in multiple myeloma.

**Significance:**

BH3 mimetics including venetoclax hold promise for treatment of multiple myeloma but rational combinations are needed to broaden efficacy. This study presents mechanistic and clinical data to support addition of pitavastatin to venetoclax regimens in myeloma. The results open a new avenue for repurposing statins in blood cancer.

## Introduction

Multiple myeloma is an incurable disease of malignant plasma cells. Treatment of multiple myeloma with combinations of proteasome inhibitors (e.g., bortezomib, carfilzomib), immunomodulatory agents, mAbs, and glucocorticoids [e.g., dexamethasone (Dex)] has resulted in improved outcomes (1). However, most patients with multiple myeloma will develop drug-resistant disease, relapse, and succumb to disease complications.

Apoptosis-targeting agents hold promise to deepen responses in multiple myeloma. The B-cell lymphoma 2 (BCL2) protein family consists of proapoptotic or antiapoptotic members that control mitochondrial apoptosis (2). The apoptosis-promoting drug class, BCL2 Homology 3 domain (BH3) mimetics, blocks the BH3 binding groove of antiapoptotic BCL2 proteins and inhibits their ability to sequester proapoptotic family members. The BCL2-specific BH3 mimetic, venetoclax, is transforming blood cancer treatment, garnering FDA approvals in chronic lymphocytic leukemia (CLL) and acute myeloid leukemia (AML; refs. 3–9). While venetoclax has not achieved regulatory approval in multiple myeloma, combination with Dex and bortezomib achieved deeper remissions over bortezomib and Dex alone corresponding to increased progression-free survival (PFS; ref. 10). However, adverse events in subsets that were not sensitive to venetoclax highlight the need for safe, alternative strategies to enhance efficacy of venetoclax in patients with multiple myeloma. MCL-1–specific BH3 mimetics, which have entered clinical trials to treat MCL-1–dependent blood cancers like multiple myeloma (11–14), may also benefit from combination agents to broaden their therapeutic utility.

Statins, mevalonate pathway inhibitors widely used to lower plasma cholesterol, are candidates for repositioning in oncology (15–18). Statins induce apoptosis in blood cancers through on-target, cancer-selective means (19–24). The rational combination of statins and venetoclax in hematologic malignancies was independently identified in three recent studies (25–27). Furthermore, CRISPR/Cas9 screens identified the mevalonate pathway as a metabolic dependency in blood cancers that sensitizes to venetoclax (28, 29). Our retrospective analysis of venetoclax clinical trial data in CLL supports the clinical relevance of these findings (25) and led to the initiation of a prospective clinical trial assessing the addition of a statin to venetoclax regimens in AML and CLL (NCT04512105), where venetoclax is standard of care.

The marked sensitivity of multiple myeloma cell lines (MMCL) to statins suggests another promising path for repurposing in blood cancers (23, 30–34). Lovastatin and simvastatin were observed to activate the integrated stress response (ISR; refs. 35, 36) and reduce MCL-1 (37, 38), promising properties for therapeutic candidates against multiple myeloma. Although efforts to translate statins into clinical combinations with chemotherapy generated unimpressive results (39–42), there exists strong mechanistic rationale to revisit statins in myeloma in light the clinical emergence of BH3 mimetics (43, 44). In addition, retrospective analyses of U.S. veterans with multiple myeloma demonstrating prolonged survival in patients undergoing treatment while concurrently on statins (45, 46) and identification of t(4;14) as a biomarker correlating with multiple myeloma cell sensitivity to statins (47) encourage a second look at repurposing statins.

We published that statins sensitize to venetoclax-mediated killing in CLL, AML, and B-cell lymphoma through a mechanism involving p53-independent upregulation of the p53 upregulated modulator of apoptosis (PUMA; ref. 25). This p53-independent mechanism imparts another advantage for repurposing statins, as p53 loss is a high-risk factor in many hematologic malignancies (48), especially multiple myeloma, and a resistance factor for BH3 mimetics (49). Here we provide preclinical and clinical evidence in multiple myeloma for the efficacy and mechanism of statin-mediated sensitization to apoptosis by BH3 mimetics against BCL2 (venetoclax) or MCL-1 (S63845).

## Materials and Methods

### Statistical Analysis

#### Cell-based and Biochemical Assays

GraphPad Prism (RRID:SCR_002798) was used to compare means using Student *t* test or ANOVA, with correction for multiple comparisons. The number of biological replicates (*n*) and details of the tests are provided in the figure legends. *P* < 0.05 was considered statistically significant and was annotated throughout as *, *P* < 0.05; **, *P* < 0.01; and ***, *P* < 0.001.

#### Retrospective Analysis of Clinical Trial Outcomes

Statistical analyses were performed to assess effect of venetoclax when it is given with and without statin on disease responses per international myeloma working group multiple myeloma response criteria. Pooled data from studies NCT01794507 and NCT02899052 were used in these analyses. Estimated response rates and corresponding 95% confidence intervals (CI) are presented. CIs of response rates were calculated by the Clopper–Pearson method. Effect of venetoclax with and without statin on disease response was statistically tested by using Cochran–Mantel–Haenszel (CMH) test. *P* values corresponding to CMH tests were presented. *P* < 0.05 was considered statistically significant and was annotated as *, *P* < 0.05; **, *P* < 0.005; and ***, *P* < 0.001. No adjustments were made for multiple comparisons. Analyses were conducted by using SAS 9.4.

### Study Approval

Research with human subjects was carried out in accordance with ethical guidelines of the Declaration of Helsinki. Peripheral blood from healthy donors was collected by the UC Irvine Institute for Clinical and Translational Sciences under Institutional Review Board (IRB) protocol HS# 2001-2058 after donors’ written informed consent; deidentified blood samples were obtained for this study under a non-human subjects determination, and used for isolation of peripheral blood mononuclear cells (PBMC). The primary multiple myeloma cell research samples used in the study have been collected under the Emory University IRB-approved protocol IRB57236. Written informed consent has been obtained from every subject prior to their participation in the study. All study related procedures have been performed as outlined in the protocol.

### Chemicals

Simvastatin (Cayman #10010344) was activated by NaOH hydrolysis per protocol (DOI: dx.doi.org/10.17504/protocols.io.j8nlkwd5xl5r/v1). Pitavastatin (AvaChem #2266), venetoclax (Active Biochem A-1231/Chemgood C-1008), S63845 (Selleck S8383/Chemgood C-1370), ISRIB (Selleck #50-136-4740), and AMG-PERK44 (MedChemExpress # HY-12661A) were dissolved in DMSO. Mevalonate (Sigma-Aldrich #90469) was resuspended with a 7:3 solution of methanol and 10 mmol/L ammonium hydroxide. TH-Z145 was prepared according to the procedures outlined in paragraphs 365 to 364 of the following patent (50). The ^1^H and ^31^P NMR of the isolated material is consistent with reported literature values (51).

### Cell Culture

Cell lines were obtained from commercial repositories (ATCC or DSMZ) and authenticated annually through short tandem repeat profiling using the University of Arizona Genomics Core; the most recent authentication date was April 6, 2023. Cell lines were tested for *Mycoplasma* infection annually; the most recent date confirmed the absence of mycoplasma on September 8, 2023. All cell lines used in study were maintained in RPMI1640, 10% FBS, 1:100 penicillin-streptomycin-glutamine (Gibco # 10-378-016) and 10 mmol/L HEPES (Corning #25-060-CI) and handled according to protocol (DOI: dx.doi.org/10.17504/protocols.io.rm7vzb6n4vx1/v1). Experiments were done using cells between 2–12 passages after thawing.

### Cell Viability

Cell viability assays were performed in 96-well format for 48 hours. Viability was determined by fluorescence of propidium iodide (PI; Life Technologies) and/or Annexin V Alexa Fluor 647 conjugate (Life Technologies) measured by the FACScalibur (Becton-Dickinson; RRID:SCR_000401), Novocyte 3000 (Agilent), or Attune NxT (Thermo Fisher Scientific). Viability of cells was quantified using FlowJo software v10.1r7 (FlowJo LLC, RRID:SCR_008520). Specific viability dyes used in figures are mentioned in figure legends. Detailed protocol is provided (DOI: dx.doi.org/10.17504/protocols.io.14egn7my6v5d/v1).

### *Ex Vivo* Human Cell Viability

Peripheral blood or bone marrow samples were filtered using 70 µm filters, diluted to 25 mL of PBS, and underlaid with lymphocyte separation medium followed by centrifugation to collect the buffy coat. Experiments were carried out in RPMI1640 with 10% FBS, Pen/Strep, and glutamine. PBMCs were treated with simvastatin in combination with venetoclax or S63845 for 48 hours, then flow cytometry was used to measure the percent viable cells in different subsets by Annexin V staining. Multiple myeloma patient cells were treated for 24 hours. Apoptosis was measured by flow cytometry after staining with anti-CD38, anti-CD45, and Annexin V to measure viability.

### Analysis of PUMA in Healthy Donor PBMCs

Healthy donor PBMCs were treated with 10 µmol/L simvastatin or with the PUMA-inducing positive control etoposide (10 µmol/L) for 20 hours. Flow cytometric assessment of PUMA was carried out according to detailed protocol (DOI: dx.doi.org/10.17504/protocols.io.q26g7yx69gwz/v1). For Western analysis of PUMA upregulation, treated cells were subjected to magnetic bead–mediated isolation using the EasySep human CD4 T cell isolation kit (Stem Cell #17952) and the EasySep human B-cell isolation kit (Stem Cell #17954). Isolated lymphocytes were processed for Western blot analysis.

### BH3 Profiling

BH3 profiling of multiple myeloma cells was performed according to the Letai lab manual on iBH3 profiling with a detailed lab specific protocol provided (DOI: dx.doi.org/10.17504/protocols.io.3byl47j4jlo5/v1). Percent depolarization caused by each BH3-only peptide was calculated as the percent difference of retained cytochrome C relative to DMSO-treated control cells. Doses of peptides for each cell line were empirically chosen based on minimal effects on mitochondrial depolarization (akin to an IC_10_ concentration).

### Western Blotting

Western blotting was conducted as described previously (25). The following antibodies were used: GAPDH (RRID:AB_561053), PARP (RRID:AB_2160739), MCL-1 (RRID:AB_10694494), BIM (RRID:AB_1030947), BID (RRID:AB_10694562), BAX (RRID:AB_2924730), BAK (RRID:AB_10828597), PUMA (RRID:AB_2797920), NOXA (RRID:AB_2798602), p-BAD (RRID:AB_10547878), BAD (RRID:AB_2062127), p-eIF2α (RRID:AB_330951), eIF2α (RRID:AB_10692650), ATF4 (RRID:AB_2616025), pAKT (RRID:AB_2315049), AKT (RRID:AB_2616025), p53 (RRID:AB_10695803), ERK (RRID:AB_390779l Cell Signaling Technology), and unprenylated RAP1A (RRID:AB_10917062) (Santa Cruz Biotechnology). Cleaved caspase-3, BCL2, and BCL-XL antibodies were from Abcam Apoptosis sampler kit (RRID:AB_514418). Chemiluminescence detected using a Nikon D700 SLR camera or the SynGene G:Box. Images were processed with autocontrast uniformly across the entire image and densitometry was performed using ImageJ software (RRID:SCR_003070).

### Overexpression and Short Hairpin RNA Knockdown

Lentiviral and retroviral production was conducted as described previously (25). Target sequences were used to generate a short hairpin RNA (shRNA) insert containing a 7-loop (TACTAGT) sequence cloned into the EZ-Tet-pLKO plasmid (Addgene plasmid # 85966, RRID:Addgene_85973). shRNA sequences were: CGCTAAATACTGGCAGGCGTT (for LACZ); GAGGGTCCTGTACAATCTCAT (for *BBC3*/PUMA). Guide RNAs targeting *BBC3* (CGCTGGGCACGGGCGACTCC) and *EIF2AK3* encoding PERK (AGATGGGAGAATTGCTGCCT, ACCATGATTTTCAGGATCCA) were cloned into lentiCRISPR v2 plasmid (Addgene plasmid # 52961, RRID:Addgene_52961). pLXSP-GSE56 (a gift from Dr. Lindsay Mayo, no RRID available) was used to generate p53 dominant negative cell lines as described previously (25).

### qRT-PCR

qPCR was conducted as described previously (25).

### Puromycin Incorporation Assay

Treated cells were pulsed with 15 minutes of puromycin (10 µg/mL; Sigma P9620). Negative controls were treated with cycloheximide (10 µg/mL; Sigma C4859) for 15 minutes prior to puromycin pulse. Sodium arsenite (50 µmol/L; Hach #104732) was used as a positive control for activation of ISR 15 minutes prior to puromycin pulse. Cells were fixed BioLegend (#421403) Foxp3-staining kit fixation buffer at room temperature for 20 minutes and permeabilized. Cells were stained with anti-puromycin AF-488 (MABE343-AF488) overnight at 4 degrees prior to flow cytometric readout. Median fluorescence intensity (MFI) of cells treated with cycloheximide is baseline for puromycin incorporation.

### Data Availability

This study includes no data deposited in external repositories. The data generated in this article are available upon request from the corresponding author.

## Results

### Retrospective Analysis of Venetoclax Clinical Trials Supports Statin Use in Multiple Myeloma

**FIGURE 1:**
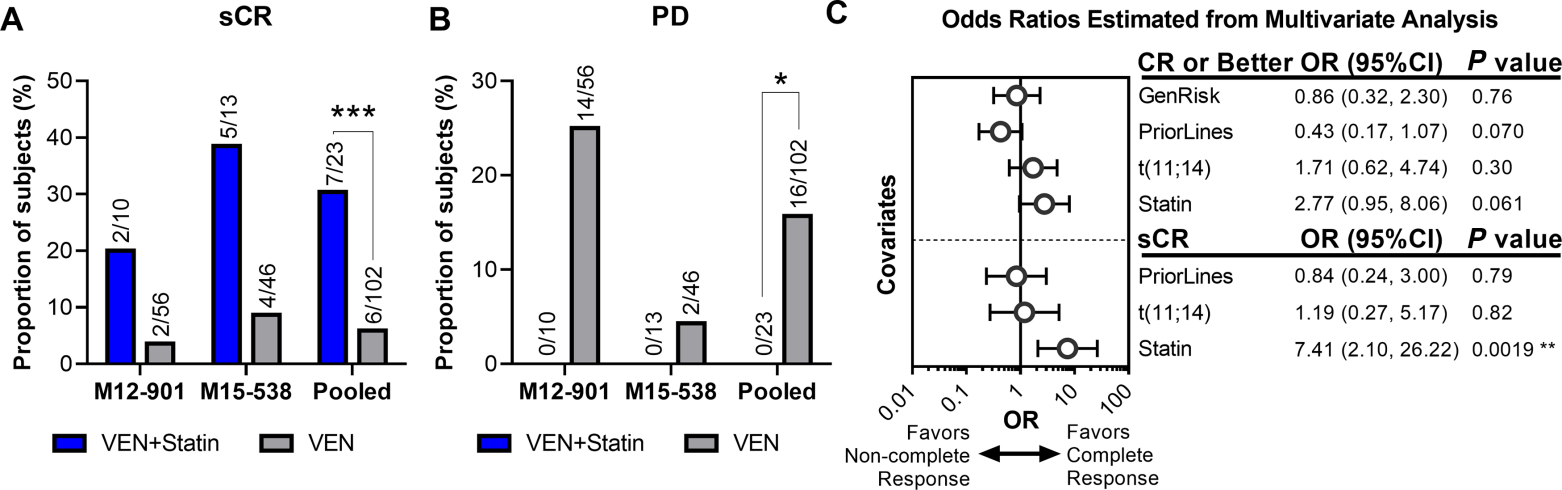
Statins significantly improve responses in a retrospective analysis of venetoclax clinical trials in R/R multiple myeloma. Results of patients with R/R multiple myeloma from venetoclax clinical trials receiving the standard therapy of Dex and a proteasome inhibitor (bortezomib for the phase Ib trial (NCT01794507 also known as M12-901) and carfilzomib for the ongoing phase II trial (NCT02899052 also known as M15-538) were pooled for *post hoc* analysis of background statin use on responses to therapy. **A,** Univariate analysis of the pooled trials assessing background statin use on response to therapy identified a significant difference in the proportion of subjects achieving sCR that is appreciated in each individual study (***, *P* < 0.001). **B,** No patient on statins experienced progressive disease (PD) in either individual study, resulting in a significant difference in the pooled analysis (*, *P* < 0.05). **C,** Multivariate analysis to evaluate known independent variables that affect patient outcomes, including prior lines of therapy, cytogenetic risk, and t(11;14) status in relation to statin use on patient responses. Statin use trended closer to support association with CR or better when accounting for prior lines of therapy, while t(11;14) and cytogenetic risk had no association with CR or better. No high-risk patients achieved sCR, thus cytogenetic risk was dropped from multivariate analysis of sCR. Neither prior lines of therapy nor t(11;14) status were associated with sCR, while statin use was strongly associated with increased rates of sCR (**, *P* < 0.01).

Statins are widely prescribed, enabling the retrospective assessment of whether background statin use influenced the venetoclax response in multiple myeloma clinical trials (NCT01794507, NCT02899052). The venetoclax phase Ib trial, M12-901 (NCT01794507), for patients with relapsed/refractory (R/R) multiple myeloma receiving the standard therapy of bortezomib and Dex enrolled 66 patients with 10 statin users. The ongoing phase II venetoclax trial, M15-538 (NCT02899052), for patients with R/R multiple myeloma treated with carfilzomib and Dex enrolled 59 patients with 13 statin users. Notably, analysis of data pooled from the two studies demonstrated significantly deeper responses by the proportion of subjects obtaining stringent complete responses (sCR; 30.4% statin users vs. 5.9% of those not on statins, *P* < 0.001; Fig. 1A; Supplementary Table S1). Moreover, no statin users had progressive disease (vs. 15.7% of non-statin users, *P* < 0.05; Fig. 1B). In this univariate analysis, statin use trended toward increased overall response rate (ORR; *P* = 0.135) and complete response (CR) or better (*P* = 0.127; Supplementary Fig. S1; Supplementary Table S1). We also conducted univariate and multivariate analysis including prior lines of therapy; t(11;14) translocation status, a biomarker for sensitivity to venetoclax/Dex in multiple myeloma (52, 53); and cytogenetic risk in accordance with International Myeloma Working Group criteria (Supplementary Tables S2–S6). In the multivariate analysis, only statin use was significantly associated with increased sCR (OR: 7.41, 95% CI: 2.10–26.22; Fig. 1C). The lack of association of t(11;14) with response is consistent with the results of multiple myeloma trials combining venetoclax/Dex with proteasome inhibitors (10, 54). Statin use trended toward being associated with CR or better response (OR: 2.77, 95% CI: 0.95–8.06) while prior lines of therapy trended away from being associated with a CR or better response (OR: 0.43, 95% CI: 0.17–1.07; Fig. 1C). There was a trend toward increased PFS among statin users in the M15-538 trial [median PFS with 95% CI for VEN = 18.9 (8.2, −); VEN + statin = 24.8 (12.4, −)] and no difference in median PFS in the M12-901 trial [VEN = 10.2 (4.3, 13.6); VEN + statin = 10.8 (5.6, 15.7)] (Supplementary Fig. S2).

### Simvastatin Sensitizes to BH3 Mimetics in MMCL Panel

**FIGURE 2:**
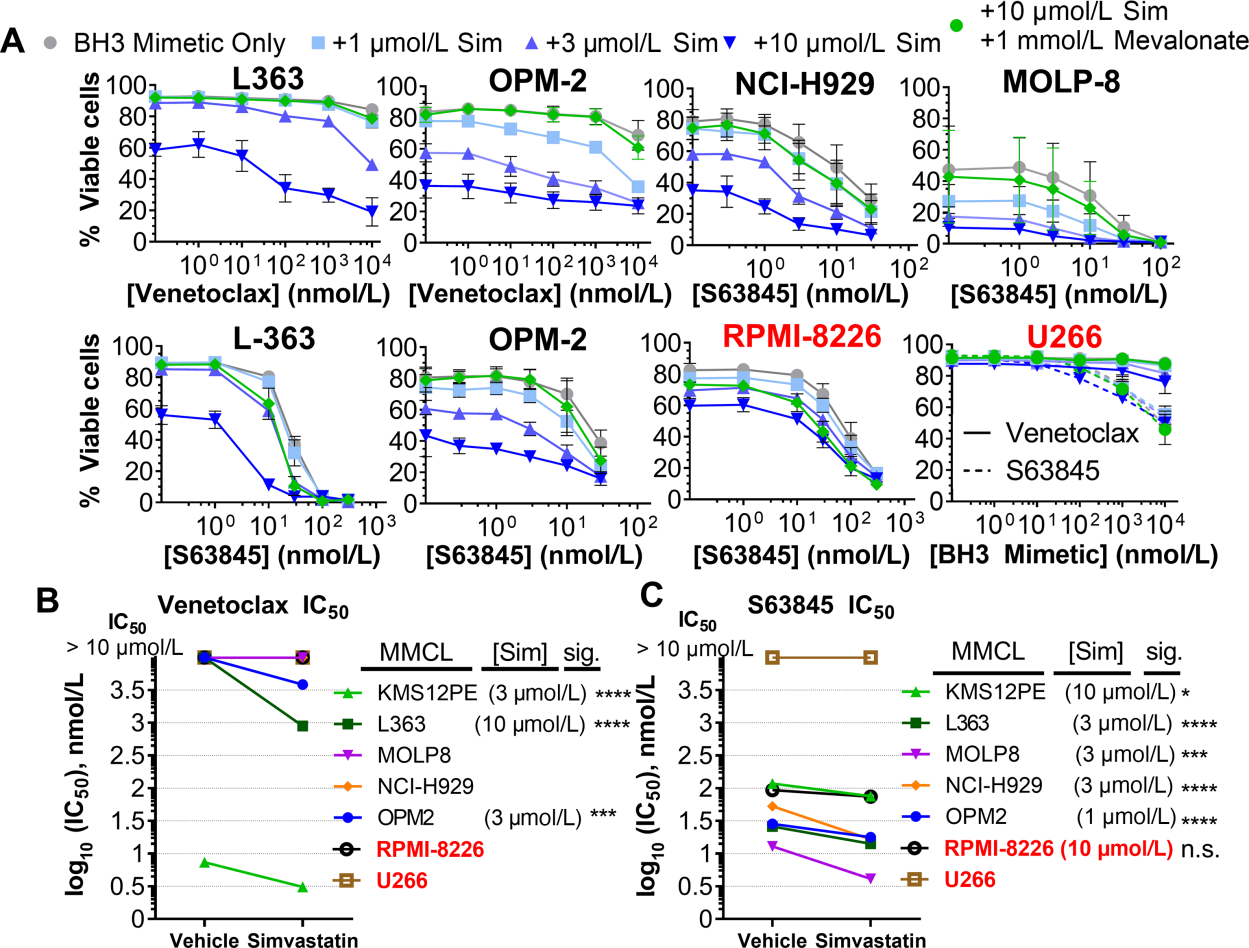
MMCLs are sensitized to BH3 mimetics by simvastatin. We assessed a panel of seven myeloma cell lines including: OPM2, L363, KMS-12-PE, NCI-H929, MOLP8, RPMI-8226, and U266. **A,** Representative plots of statin-sensitive (black) MMCLs are shown alongside statin insensitive (red). The initial screen used PI exclusion to determine viability by flow cytometry. Rescue with 1 mmol/L mevalonate was demonstrated at the highest concentration of simvastatin to demonstrate on-target activity. Of note, statin-mediated killing of RPMI-8226 is not fully rescued by mevalonate, perhaps due to inefficient cellular uptake. *n* = 3, error bars indicate SD. **B,** IC_50_ of venetoclax or **C,** S63845 at concentrations of statins necessary to significantly shift the IC_50_. Significance (*P* value <0.05) was determined by extra sum-of-squares F test for differences in log IC_50_. Achieving significance was the classification determinant for statin-sensitive and statin-insensitive cell lines. *, *P* < 0.05; **, *P* < 0.01; *** , *P* < 0.001; ****, *P* < 0.0001 of Bonferroni-adjusted *P* values.

To understand the mechanism of statin/BH3 mimetic combinations in multiple myeloma, we characterized the sensitivity of a panel of seven MMCLs including three (KMS12PE, U266, MOLP8) harboring t(11;14) translocation, and two (OPM2, NCI-H929) harboring t(4;14) translocations. For our initial screen we used simvastatin, the statin compound that was the primary agent applied in our leukemia study (25). We evaluated cell viability after 48 hours treatment with combinations of simvastatin and venetoclax or the MCL-1 inhibitor S63845 (Fig. 2A; Supplementary Fig. S3). Consistent with the consensus that multiple myeloma cell survival is maintained primarily by MCL-1, venetoclax achieved 50% killing (IC_50_) at concentrations under 10 µmol/L in only one MMCL (KMS12PE; Fig. 2B; Supplementary Table S7), whereas S63845 achieved an IC_50_ in the low to mid-nanomolar range (10–100 nmol/L) for all but one line tested (U266; Fig. 2C; Supplementary Table S7).

Simvastatin alone at concentrations of 10 µmol/L or lower reduced viability in all MMCLs except U266 (Fig. 2A; Supplementary Fig. S3A). In combination, increasing concentrations of simvastatin significantly shifted the BH3 mimetic IC_50_ at the indicated doses (Fig. 2B and C). With venetoclax, simvastatin reduced the IC_50_ below 10 µmol/L in three MMCLs, while with S63845, simvastatin significantly shifted the IC_50_ in five MMCLs. The sensitization by 10 µmol/L simvastatin was completely reversed by supplementing cell media with mevalonate, demonstrating that statin-mediated apoptotic sensitization is an on-target effect of HMG-CoA-reductase inhibition (Fig. 2A; Supplementary Fig. S3). Notably, simvastatin did not sensitize to BH3 mimetics in RPMI-8226 and U266. This cell line variability aligns with our previous studies of leukemia and lymphoma lines (25), highlighting biological heterogeneity that influences susceptibility to statin-mediated apoptotic sensitization. Simvastatin showed evidence of synergy with venetoclax and/or S63845 using two different algorithms, the BLISS independence model that defines synergy by the multiplicative survival principal and highest single agent model that defines synergy as excess over the highest single agent (Supplementary Table S8). Simvastatin combinations with venetoclax were synergistic in OPM2, L363, and KMS12PE whereas with S63845, NCIH929, OPM2, and L363 demonstrated synergy and MOLP8 and KMS12PE favored additivity (exemplary topology in Supplementary Fig. S3B).

### PUMA Protein Upregulation Correlates with Statin Sensitivity in MMCL and is p53 Independent

**FIGURE 3:**
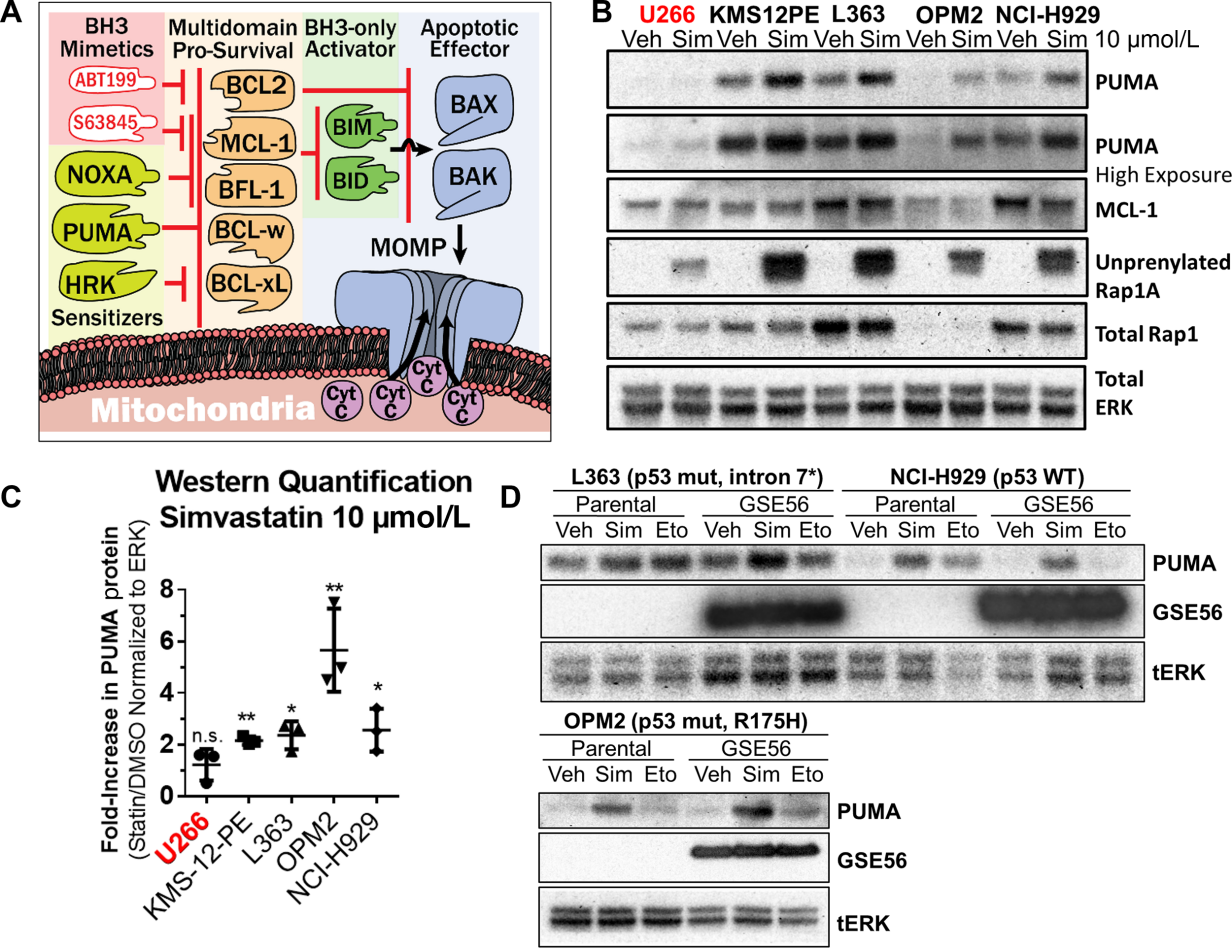
Simvastatin induces p53-independent PUMA upregulation. **A,** A BCL2 family model figure depicting protein interactions and how BH3 mimetics can cooperate with apoptotic sensitizers. **B,** A representative Western blot analysis of statin-insensitive (red) and statin-sensitive (black) MMCLs treated for 16 hours with 10 µmol/L simvastatin. *n* = 3. **C,** Quantification by densitometry of PUMA upregulation. Mean ± SD, *n* = 3, significance (α<0.05) determined by one-sample *t* test of log-transformed fold changes against the value of 0. *, *P* < 0.05; **, *P* < 0.01. **D,** Representative (*n* = 3) Western blots showing that PUMA upregulation by 10 µmol/L simvastatin is not dependent on the transcriptional activity of p53. Expression of the p53 dominant negative, GSE56, was accomplished in three MMCLs, L363, NCI-H929, and OPM2 wherein the basal p53 status is mutated [*L363 cells have a point mutation in exon7/intron 7 splicing junction (base C782G) that impairs splicing of intron 7], wild-type, and mutated (R175H), respectively. GSE56 expression was confirmed with a rodent-specific p53 antibody (Cell Signaling Technology# 32532). Activation of p53 using 10 µmol/L of the topoisomerase inhibitor, etoposide, was used as a positive control to confirm blockade of p53 activation of PUMA upregulation.

To directly assess apoptotic sensitization, we employed dynamic BH3 profiling to measure the statin-mediated change in cellular proximity to cytochrome C release termed “priming” (Supplementary Fig. S4A). Using the BH3 peptide of BIM, a promiscuous antiapoptotic binder and activator of BAX and BAK (Fig. 3A), we verified that simvastatin induces overall apoptotic priming in tested MMCLs (Supplementary Fig. S4B), except U266 corresponding to viability assays results (Fig. 2A).

In accordance with this apoptotic priming and prior findings (25), simvastatin upregulated the proapoptotic PUMA protein in all cell lines showing shifted BH3 mimetic IC_50_s, henceforth referred to as “statin-sensitive” MMCLs, after 16 hours treatment with 10 µmol/L simvastatin (Fig. 3B and C). In all MMCLs, statin treatment induced appearance of unprenylated RAP1A confirming blockade of mevalonate pathway dependent protein geranylgeranylation. Notably, OPM2 demonstrated the greatest magnitude of PUMA upregulation (Fig. 3C), in agreement with the lower simvastatin concentrations required to shift BH3 IC_50_s (Fig. 2A). Under the same conditions, the *BBC3* transcript encoding PUMA was upregulated compared with DMSO control treatment (Supplementary Fig. S4C); however, significant transcript upregulation was also apparent in cell lines wherein statin treatment did not shift BH3 mimetic IC_50_s.

We generated cell lines overexpressing a dominant-negative p53 oligomerization domain fragment, GSE56 (55), and assessed PUMA upregulation in response to statins or to the DNA-damaging agent etoposide as a control to induce p53-dependent PUMA expression (56). GSE56 blocked etoposide-mediated PUMA upregulation, but not simvastatin-mediated PUMA upregulation (Fig. 3D). Expression of GSE56 did not affect statin sensitization to apoptosis compared with their parental cell line (Supplementary Fig. S4D–S4F) but did induce resistance to S63845 in NCIH929 and L363 MMCLs (Supplementary Fig. S4D–S4F). OPM2 did not respond to etoposide due to p53 mutation R175H, a mutation of p53 known to inhibit p53 family members p63 and p73 (57–61). However, OPM2 upregulated PUMA expression in response to 10 µmol/L simvastatin (Fig. 3D), suggesting that p53 family proteins are not involved in statin-mediated PUMA upregulation.

We assessed selectivity of the statin apoptotic sensitization using healthy donor PBMCs. Following 48 hours treatment, BH3 mimetics alone were cytotoxic to CD19^+^ B cells, yet simvastatin did not significantly potentiate killing (Supplementary Fig. S5A). This toxicity to peripheral B cells is known (62–64) and many B-cell–depleting agents are tolerable in patients. Simvastatin did not potentiate the killing of CD4^+^ or CD8^+^ T cells or CD3^−^/NK56+ natural killer cells, consistent with patient studies (65). PUMA protein was measured by intracellular staining of cocultures (Supplementary Fig. S5B) and by Western blot analysis of magnetically isolated CD4^+^ and CD19^+^ lymphocytes (Supplementary Fig. S5C). Unlike etoposide, simvastatin did not upregulate PUMA in healthy PBMCs, highlighting cancer selectivity of apoptotic sensitization by statins.

### Loss of Protein Geranylgeranylation Sensitizes to BH3 Mimetics

We previously observed the apoptotic effects of statins in leukemia and lymphoma were due to depletion of intermediary isoprenoids, farnesyl pyrophosphate (FPP) or geranylgeranyl pyrophosphate (GGPP) that are necessary for protein prenylation, not cholesterol (25). Therefore, we tested whether prenyltransferase inhibitors, FTI-277 and GGTI-298, or the geranylgeranyl diphosphate synthase inhibitor TH-Z145 [a proxy to inhibit all protein geranylgeranylation including from geranylgeranyltransferase (GGTase) II and the newly discovered GGTase III (66)] could sensitize to BH3 mimetics (Supplementary Fig. S6A). Apoptotic sensitization from these drugs varied across all statin-sensitive MMCLs, indicating the involvement of diverse prenylation substrates in the statin apoptotic sensitization mechanism. However, frequent sensitization to BH3 mimetics was observed with GGTI-298 and TH-Z145 treatment, indicating the importance of protein geranylgeranylation in the sensitization to apoptosis (Supplementary Fig. S6B–S6F).

### Pitavastatin is a More Potent Statin Compound

**FIGURE 4:**
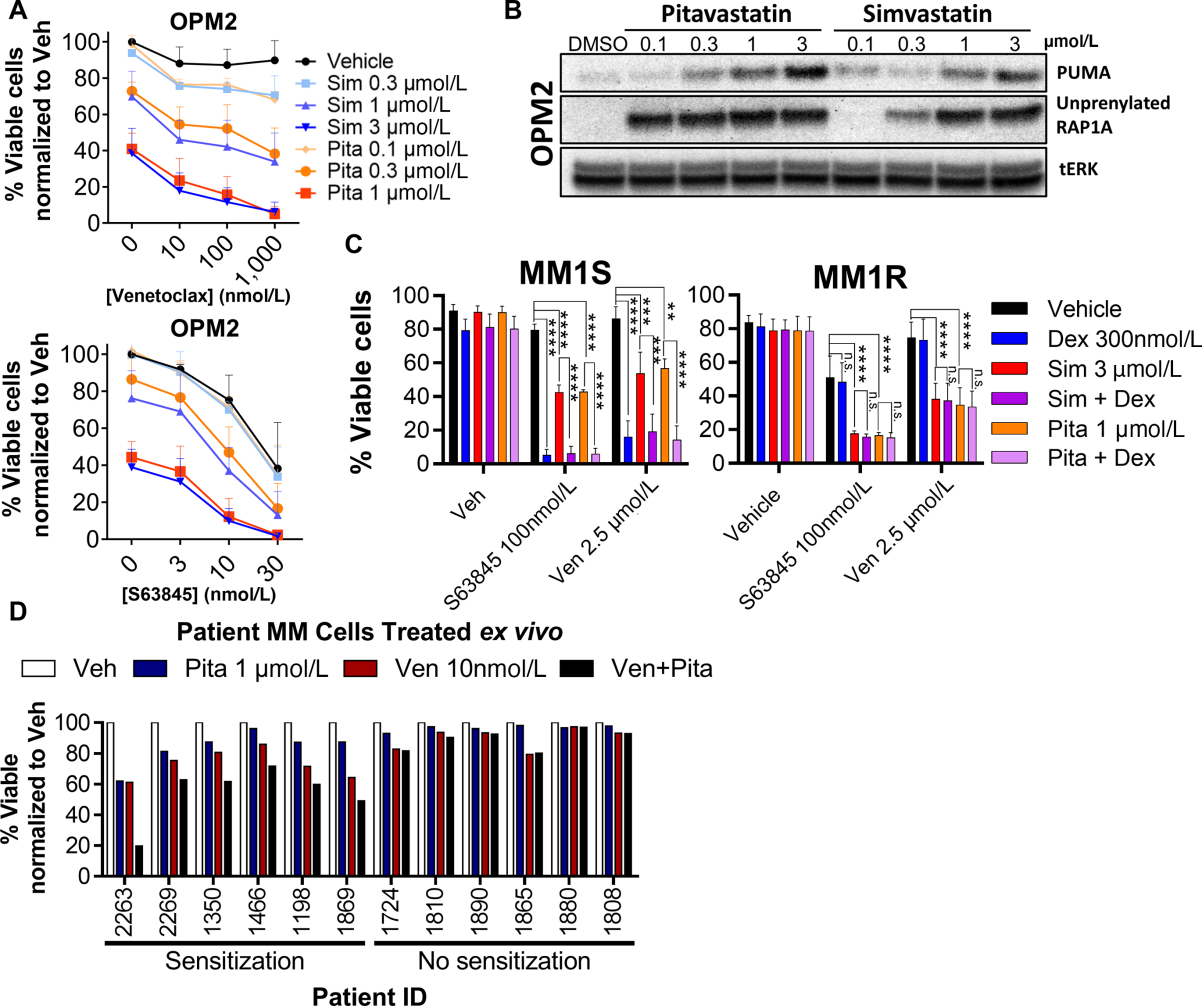
Pitavastatin is three times more potent than simvastatin in cell-based assays of apoptosis and biomarker responses, and increases apoptosis in combination with venetoclax in a subset of patient samples treated *ex vivo*. **A,** Cell Titer Glo was used to assess viability in statin/BH3 mimetic combinations after 48 hours; % viable cells was normalized to vehicle. Mean ± SD, *n* = 3. **B,** Pitavastatin increases PUMA and blocks prenylation of RAP1A at concentrations lower than simvastatin. **C,** Results of a glucocorticoid resistance cell line models MM.1S (sensitive) and MM.1R (resistant) after 48 hours treatment. Mean ± SD; *n* = 3; *, *P* < 0.05; **, *P* < 0.01; ***, *P* < 0.001; ****, *P* < 0.0001, one-way ANOVA with Tukey post-test correction for multiple comparisons. **D,** Buffy coats from multiple myeloma patient bone marrow aspirates were treated *in vitro* with 1 µmol/L pitavastatin and 10 nmol/L venetoclax for 24 hours before staining with CD45, CD38, and Annexin V to assess viability.

Pitavastatin has a pharmacokinetic profile that is more suitable for drug repurposing in oncology (42, 67), including prolonged plasma half-life (10–12 hours) and higher plasma concentration levels (100–500 nmol/L) at standard doses (68–71). Pitavastatin was three times more potent on a molar basis than simvastatin at inducing MMCL death (Fig. 4A). Similarly, we observed that in MMCLs, pitavastatin is approximately 3-fold more potent than simvastatin at inducing PUMA upregulation and accumulation of unprenylated RAP1A (Fig. 4B; Supplementary Fig. S7A). The general apoptotic sensitization by statins holds for pitavastatin at mid-nanomolar concentrations, as 300 nmol/L pitavastatin primed OPM2 for apoptosis by the BIM, PUMA, BAD, and MS1 peptides (Supplementary Fig. S7B).

### Statins Increase Venetoclax Cytotoxicity in a Dex-resistant MMCL

Multiple myeloma clinical trials are assessing venetoclax in combination with Dex, a mainstay multiple myeloma therapy with well-characterized apoptosis-sensitizing properties (72). We compared the apoptosis-promoting effects of simvastatin and pitavastatin with the effects of Dex in a MMCL model of glucocorticoid resistance. MM.1S and MM.1R are isogenic cell lines differing in their expression of the glucocorticoid receptor (73) that imparts Dex resistance in MM.1R. As expected, Dex enhanced apoptosis induced by BH3 mimetics in MM.1S, but not in MM.1R cells (Fig. 4C). However, statins increased BH3 mimetic induced apoptosis in both MM.1S and MM.1R. The glucocorticoid-induced leucine zipper (GILZ) protein is posited to be the apoptotic determinant for Dex cytotoxicity in multiple myeloma (74). MM.1R lost the ability to upregulate GILZ in response to Dex; however, pitavastatin induced GILZ expression in both MM.1R and MM.1S without hindering the Dex response (Supplementary Fig. S8). Thus, statins may be a useful adjunct therapy in the setting of Dex resistance.

### MM Patient Cells can be Sensitized to BH3 Mimetics by Pitavastatin

We treated patient bone marrow buffy coat cells *ex vivo* with venetoclax and pitavastatin for 24 hours to assess sensitization to venetoclax cytotoxicity in patient-derived multiple myeloma cells. The buffy coat layer includes bone marrow stromal cells which support survival of the cocultured multiple myeloma cells (identified as CD38^+^/CD45lo) *ex vivo*. Pitavastatin alone (1 µmol/L) reduced the viability of five samples. In six patient samples out of 12, pitavastatin sensitized to venetoclax-mediated killing (Fig. 4D) including two of three samples with t(11;14) translocation, one of two with t(4;14), two of seven with chromosome 1q amplification, and two of five with chromosome 17p-deletion implicating p53 loss (Supplementary Table S9). Thus, statins can sensitize multiple myeloma cells from a subset of patients to BH3 mimetic killing, including some with loss of p53 function.

### PUMA Upregulation Contributes to Statin Sensitization

To test the functional role of statin-induced PUMA, we generated L363 cell clones with CRISPR/Cas9-mediated knockout of the *BBC3* gene (Supplementary Fig. S9A). PUMA knockout clones, as confirmed by western, consistently showed partial rescue from 10 µmol/L pitavastatin (Supplementary Fig. S9B). One out of three knockout clones demonstrated partial rescue from combination of 1 µmol/L pitavastatin and 10 µmol/L venetoclax, and two clones exhibited partial rescue from combination of 1 µmol/L pitavastatin and 30 nmol/L S63845, highlighting clonal variability in proximity to apoptosis.

Circumventing the need to evaluate single-cell clones with potential compensatory effects, we assessed the PUMA contribution to apoptotic sensitization with a doxycycline-inducible shRNA approach. The induced knockdown of PUMA to levels comparable to non–pitavastatin-treated L363 cells (Supplementary Fig. S9C) resulted in partial rescue of viability in cells treated with venetoclax plus pitavastatin (Supplementary Fig. S9D). These data provide additional evidence that PUMA upregulation is a contributing factor in statin-mediated apoptotic sensitization but indicate that other mechanisms exist.

### Pitavastatin Activates the ISR

**FIGURE 5:**
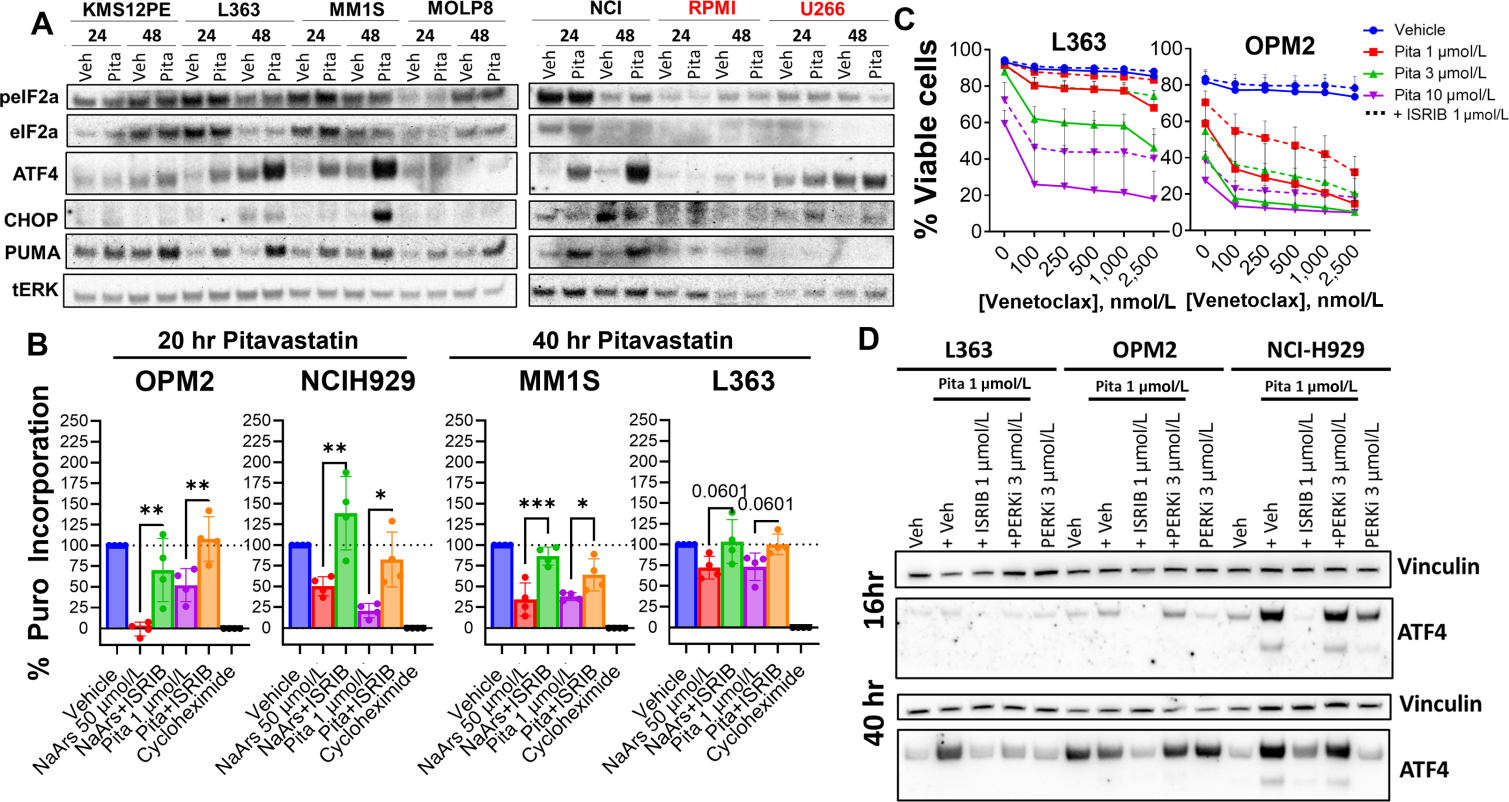
Activation of stress pathways is induced by pitavastatin and the ISR inhibitor, ISRIB, partially rescues from statin-mediated sensitization of BH3 mimetics. **A,** ATF4 and stress activated factors were assessed across our panel of cell lines by Western blot analysis, including five statin sensitive, and two statin insensitive MMCL (RPMI-8226 and U266). **B,** Puromycin incorporation was measured by flow cytometry as a marker for active translation, and was assessed after 15 minutes of puromycin exposure. MMCLs were treated with 20 or 40 hours of pitavastatin or pitavastatin/ISRIB. Fifteen minutes prior to puromycin exposure, control wells were treated with sodium arsenite (NaArs), NaArs/ISRIB, or cycloheximide. MFI of cycloheximide-treated cells served as background fluorescence and was subtracted from the MFI of all other samples. Data are presented relative to vehicle treated cells. Significance of ISRIB rescue was determined by one-way ANOVA and multiple comparisons were corrected by Holm-Sidak multiple comparisons test, *, *P* < 0.05; **, *P* < 0.01; ***, *P* < 0.001. **C,** AnnexinV-PI viability assays of L363 and OPM2. ISRIB rescues from the apoptosis induced by venetoclax and pitavastatin in L363 and OPM2, *n* = 3. **D,** Western blot analysis of ATF4 upregulation induced by pitavastatin after 16 or 40 hours for the purpose of assessing the effect of AMG-PERK44 (PERKi) in L363, OPM2, and NCI-H929. Upregulation of ATF4 is delayed in L363 relative to the t(4;14) MMCLs, OPM2 and NCI-H929, consistent with puromycin incorporation results. PERK inhibition blocks ATF4 upregulation in L363, but not OPM2 and NCI-H929.

Two groups have reported that statin treatment of multiple myeloma cells triggers the ISR (36, 47), with one report showing preferential ISR activation in cells with the t(4;14) translocation (47). Indeed, Western blots showed an increase in the hallmark ISR transcription factor, ATF4, in pitavastatin treated MMCLs with t(4;14) (OPM2, NCI-H929) and to varying degrees in t(11;14) (MOLP8) and other genetic backgrounds (L363, MM.1S) in a time-dependent manner (Fig. 5A). MMCLs resistant to statin sensitization (RPMI-8226, U266) did not show ATF4 increases.

We did not observe consistent appearance of hallmark ISR markers, phosphorylated eIF2α (p-eIF2α) or expression of CHOP, across MMCLs at the timepoints measured (Fig. 5A), likely due to the transient nature of p-eIF2α (75) and the non–ISR-dependent regulation of CHOP (76, 77). Instead, we assayed the functional outcome of the ISR by measuring protein translation using a flow cytometry–based puromycin incorporation assay (78). In response to pitavastatin, statin-sensitive MMCLs displayed a time-dependent inhibition of protein synthesis (Fig. 5B; Supplementary Fig. S10A). We used a chemical inhibitor of the integrated stress response (ISRIB) that allosterically blocks p-eIF2α from competitively inhibiting eIF2B (79). ISRIB reversed the statin-mediated reduction of protein translation in statin-sensitive MMCLs (Fig. 5B; Supplementary Fig. S10A) similarly to its effect on the sodium arsenite positive control, suggestive of cryptic increase of p-eIF2α by statins (80). ISRIB partially rescued pitavastatin-treated cells from sensitization to venetoclax-mediated apoptosis (Fig. 5C; Supplementary Fig. S10B–S10E). Notably, sensitization to S63845 was mostly unaffected by ISRIB. In two statin-sensitive AML cell lines (25), OCI-AML3 and MOLM13, pitavastatin did not induce ATF4 and ISRIB did not affect sensitization to venetoclax, suggesting statin-mediated activation of the ISR is multiple myeloma specific (Supplementary Fig. S11).

Lovastatin treatment reportedly halts immunoglobulin trafficking in multiple myeloma cells and activates the classically PERK-mediated unfolded protein response (36). We used CRISPR/Cas9 gene editing to generate L363 cell clones lacking PERK (Supplementary Fig. S12A and S12B) and confirmed the absence of ATF4 induction following treatment with a positive control stimulus, tunicamycin (Supplementary Fig. S12A). The PERK-deficient L363 clones failed to upregulate ATF4 in response to pitavastatin (Supplementary Fig. S12B) and ISRIB no longer rescued from the effect of pitavastatin and venetoclax (Supplementary Fig. S12C). In accord, the PERK inhibitor AMG-PERK44 (81) blocked ATF4 induction by pitavastatin in L363 cells (Fig. 5D) at a concentration (3 µmol/L) that suppresses tunicamycin-mediated ATF4 induction (Supplementary Fig. S12D). However, AMG-PERK44 did not prevent ATF4 induction by pitavastatin in OPM2 and NCIH929 (Fig. 5D), suggesting alternative mechanisms of ISR activation in the t(4;14) cell lines.

### NOXA Upregulation is Mediated by ISR Activation and Contributes to Statin Sensitization to Venetoclax

**FIGURE 6:**
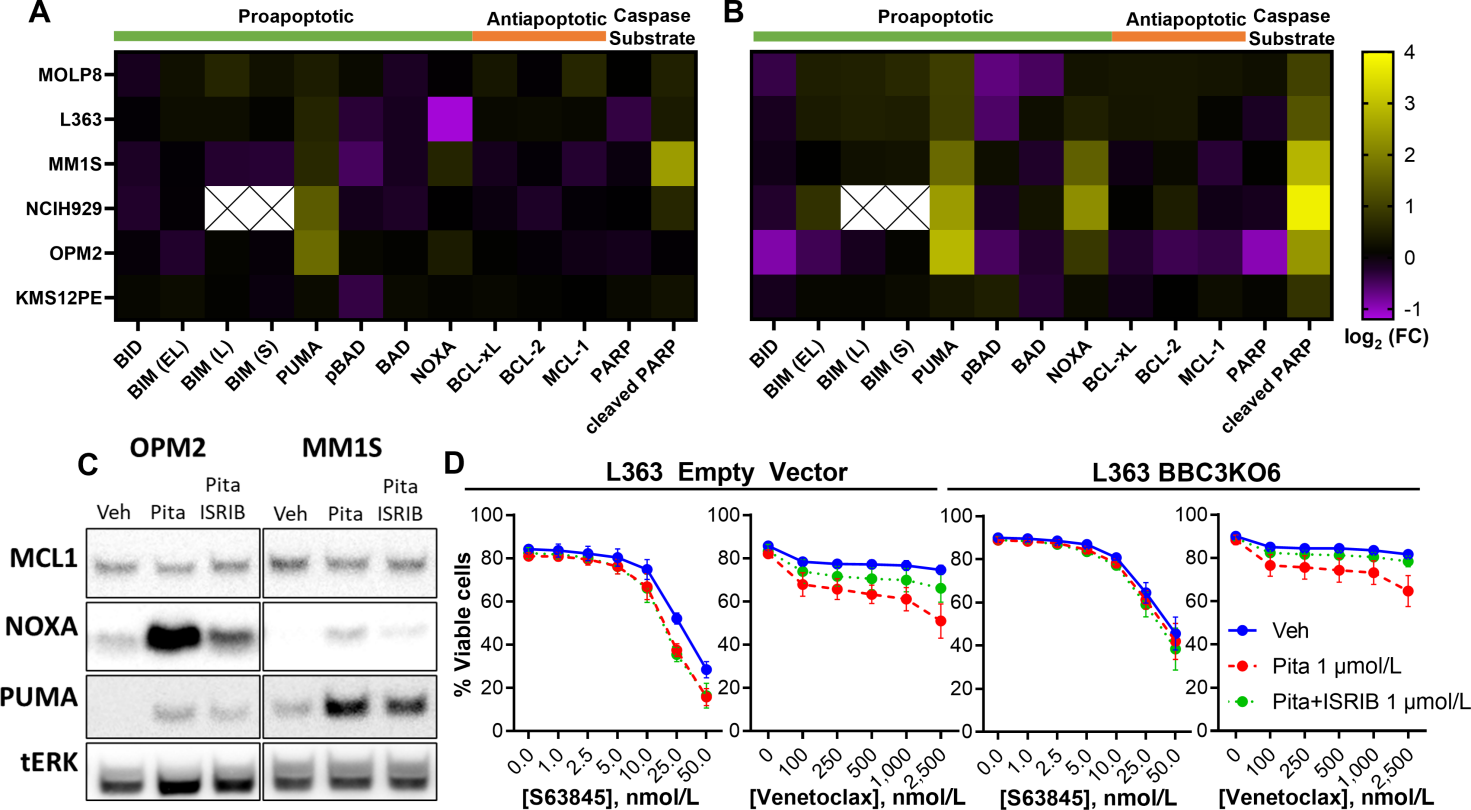
Pitavastatin-mediated activation of the ISR induces NOXA to sensitize to venetoclax separately from PUMA. Heat map summary of BCL2 family Western blot screen across all statin-sensitive cells (NCI-H929, MM.1S, L363, OPM2, MOLP8, and KMS12PE) for 16 (**A**) and 40 hours (**B**) treatments with 1 µmol/L pitavastatin. log_2_ transformed mean fold change of three replicates of each MMCL are plotted on a double gradient scale. **C,** Western blot analysis of the t(4;14) OPM2 and non-t(4;14) MM1S treated with 1 µmol/L pitavastatin or pitavastatin/ISRIB. NOXA upregulation is partially blocked by ISRIB, but PUMA is mostly ISR independent. Representative of *n* = 2 for tested cell lines. **D,** The apoptosis sensitizing effects of pitavastatin are blocked in *BBC3* (PUMA) knockout L363 cell line treated with ISRIB in a 40-hour Annexin V-PI viability assay, *n* = 4.

To identify the ISR-dependent contributor of apoptotic sensitization, we measured expression of members of the BCL2 family by Western blot analysis 16 and 40 hours after pitavastatin treatment of statin-sensitive MMCLs. At the 16-hour timepoint only PUMA showed consistent upregulation (Fig. 6A). However, at 40 hours (Fig. 6B), we observed increases in expression of NOXA, another BH3-only sensitizer that has been linked to the ISR and ATF4 (82). Notably, at this timepoint, we also detected PARP cleavage (Fig. 6B), a caspase substrate that serves as a hallmark for apoptosis. Cotreatment with ISRIB largely prevented the increase in NOXA, with minimal effect on PUMA (Fig. 6C). Using a Cas9-inducible MMCL (KMS18), we observed that NOXA knockout results in the near complete loss of sensitivity to venetoclax (Supplementary Fig. S13A). PUMA knockout in this same cell line resulted in partial rescue from apoptotic sensitization by pitavastatin.

**FIGURE 7:**
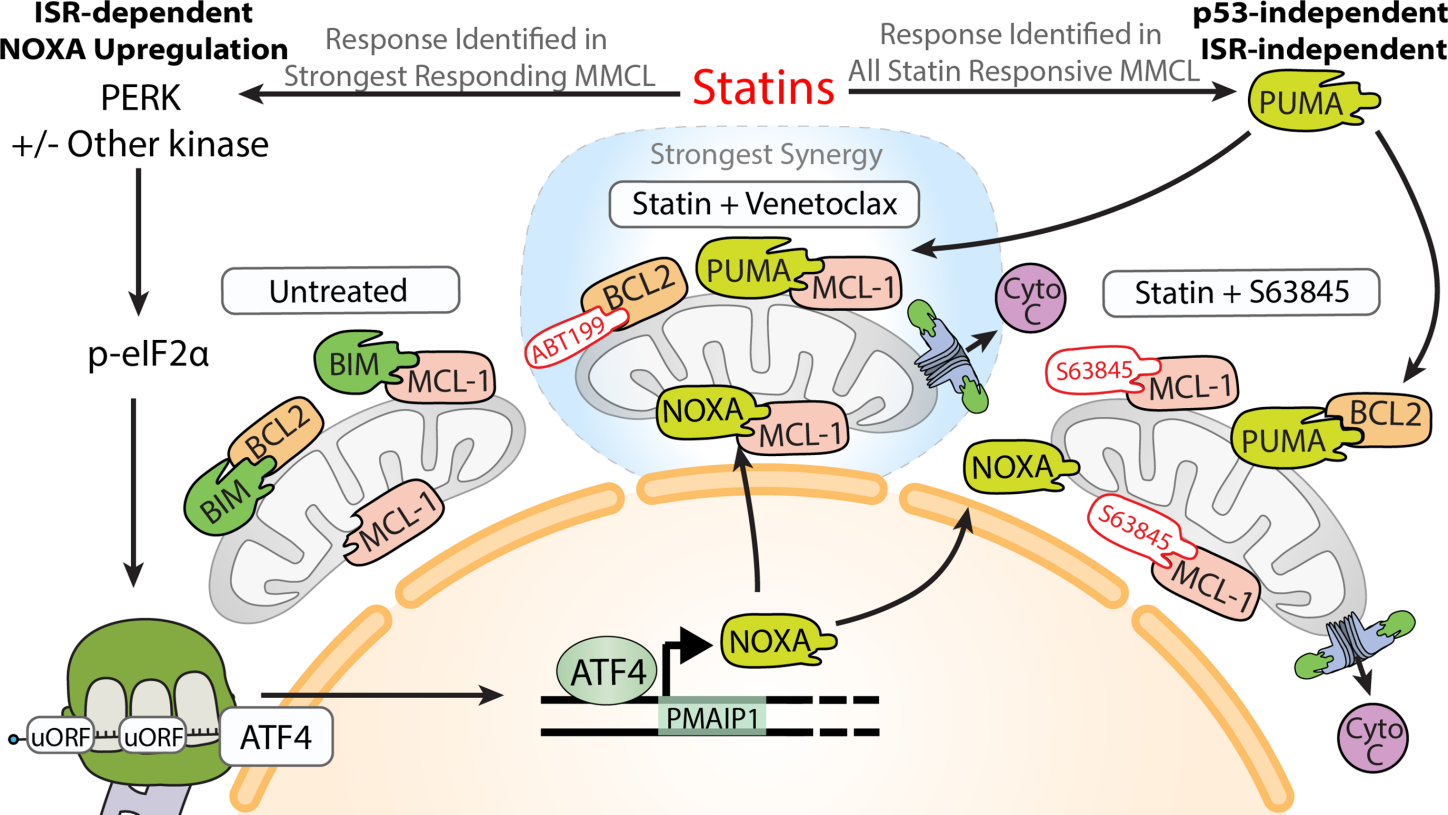
Statins induce two independent responses in multiple myeloma. As observed in AML, DLBCL, and now multiple myeloma, statins upregulate PUMA in a p53-independent manner. PUMA can contribute to BH3 mimetic killing by promiscuously binding prosurvival BCL2 family members. Activation of the integrated stress response is a multiple myeloma–specific statin response that influences apoptosis. Through a currently undefined mechanism, statins reduced general translation and activate the translation of ATF4. ATF4 is a master mediator of the ISR. One such output of ATF4 is upregulation of NOXA. NOXA can be partially reduced with ISRIB which results in the partial rescue from statin-mediated apoptotic sensitization to venetoclax. The MCL-1 selective activity of NOXA promotes sensitivity to venetoclax. However, this property also makes it a direct competitor with S63845. As such, ISRIB did not rescue from S63845 and statin-mediated killing.

The addition of ISRIB to an L363 PUMA knockout clone completely rescued the venetoclax sensitizing effects of statins, while PUMA knockout alone was sufficient to block the effect of S63845 (Fig. 6D). Doxycycline-inducible shRNA against PUMA confirmed the roles of PUMA upregulation and ISR activation in statin-mediated venetoclax sensitization in L363 (Supplementary Fig. S13B). These results bolster a mechanism wherein statins more strongly overcome resistance to BCL2 inhibition relative to MCL-1 inhibition (Fig. 7).

## Discussion

The mevalonate pathway is a targetable dependency in blood cancer for which FDA-approved agents already exist, statins (44). Our results indicate the cancer-specific, proapoptotic effects of statins may be leveraged by combining with BH3 mimetics. Statins sensitized MMCLs to BH3 mimetics, including in a model of Dex resistance, and patient multiple myeloma cells to venetoclax. The leading clinical candidate, pitavastatin, was more potent and induced cell death and biomarker responses at clinically achievable concentrations, compared with prior candidates (39–41). Previously we presented preliminary analyses of phase I trials showing that statin use trended toward increased ORR and very good partial response (VGPR) among patients with R/R multiple myeloma treated with venetoclax/Dex/bortezomib (M12-901) and the t(11;14) translocation subset analysis in patients with R/R multiple myeloma treated with venetoclax/Dex (M13-367; NCT01794520; ref. 83). In our current study, the clinical utility of statins and venetoclax is supported by pooled, retrospective analysis of venetoclax clinical trials wherein statin use correlated with significantly deeper responses (increased sCR, absence of progressive disease). These retrospective datasets likely underestimate the potential benefit of pitavastatin as the statin drugs more commonly prescribed for cholesterol control are not optimized for oncology.

Statin-sensitive MMCLs shared the property of upregulating PUMA protein in a p53-independent manner as observed in AML (25). Statins uniquely activate the ISR in several of the most sensitive MMCLs. ISRIB enabled identification of statin-mediated, ISR-induced reduction of protein translation, observation of differential rescue of statin sensitization to venetoclax versus S63845, and determination of the ISR-responsive apoptotic factor, NOXA, in MMCLs. These results support a mechanistic model where statins trigger distinct and parallel proapoptotic signals via PUMA and the ISR/NOXA axis in MMCLs (Fig. 7). This model explains the superior combination effects of statins with venetoclax, because NOXA induction is mechanistically redundant with chemical MCL-1 inhibition. In absence of a NOXA response, the statin-mediated PUMA response still acts to sensitize MMCLs to BH3 mimetics. While t(4;14) MMCLs and patient samples exhibit notable sensitivity to statins (47), our data extend statin utility to non-t(4;14) multiple myeloma by combination with BH3 mimetics where PUMA, NOXA, and ATF4 are biomarkers for statin sensitivity.

How statins activate the ISR is not fully understood. The insight that lovastatin triggers markers of the unfolded protein response through a mechanism involving loss of RAB geranylgeranylation hindering immunoglobulin light chain trafficking (36), aided in uncovering the role of PERK in L363 cells. However, ATF4 induction in t(4;14) MMCLs was not blocked by chemical PERK inhibition. Future studies should address the roles of different eIF2α kinases with the considerations that multiple kinases may be involved, especially at high statin concentrations, and responses at clinically achievable doses should be prioritized for clinical translation.

Unlike reports that galvanized the initiation of high-dose simvastatin clinical trials in multiple myeloma (37, 38), we did not observe consistent MCL-1 reduction to statin treatment. Several differences may explain this discrepancy including statin concentration (1 µmol/L pitavastatin vs. 30 µmol/L lovastatin), longer timepoints (16–40 hours vs. 48–96 hours), and cell line heterogeneity. The understanding gained in our study adds to this body of work by implicating NOXA upregulation as the potential mediator of MCL-1 decrease (84). Identification of pitavastatin, with a half-life (12 hours) that aligns with the early upregulation of PUMA and NOXA, revitalizes the hopes undercut by the short half-life of simvastatin/lovastatin (2–3 hours) and the time required to reduce MCL-1. Our larger panel of MMCLs captured decreased MCL-1 in OPM2 and MM.1S at 40 hours (Fig. 6B), but the generalizability and functional importance of this finding is uncertain.

Key questions remain regarding the mechanism of statin-induced apoptotic sensitization in blood cancers. To date, we have not identified the transcriptional regulators of PUMA upregulation. Further research is needed to better define the signaling mechanisms and gene regulatory networks that connect mevalonate pathway inhibition to the ISR, PUMA, and NOXA.

## Supplementary Material

Figure S1 Figure S1 contains additional data from retrospective analysis of venetoclax clinical trials in MM

Figure S2 Supplementary Figure 2 contains PFS data from venetoclax clinical trials in MM

Figure S3 Supplementary Figure 3 contains additional viability assay data and synergy contour plots

Figure S4 Supplementary Figure 4 contains BH3 profiling data, and viability assays in cells expressing p53 dominant negative

Figure S5 Supplementary Figure 5 contains viability data and PUMA expression data from PBMCs

Figure S6 Supplementary Figure 6 contains viability data from cells treated with the GGPP synthase inhibitor TH-Z145

Figure S7 Supplementary Figure 7 contains data comparing pitavastatin with simavastatin in western blots (panel A) and BH3 profiling data with pitavastatin (panel B).

Figure S8 Supplementary Figure 8 contains data on GILZ mRNA expression in MM1S and MM1R cells

Figure S9 Supplementary Figure 9 contains data on L363 cells with PUMA (BBC3) knockout or inducible knockdown.

Figure S10 Supplementary Figure 10 contains additional data on ISR-dependent changes in protein synthesis rates and viability.

Figure S11 Supplementary Figure 11 presents data showing that pitavastatin does not activate ISR in AML cell lines.

Figure S12 Supplementary Figure 12 contains additional data on the role of PERK in statin-induced ISR in MM cell lines.

Figure S13 Supplementary Figure 13 presents data on Cas9-mediated NOXA and PUMA deletion in KMS18 cells (panel A) and viability assays in cells with PUMA knockdown and ISRIB treatment (panel B).

Table S1 Supplementary Table S1 presents statistical analysis of pooled clinical trial data on R/R MM patient response to venetoclax treatment, comparing statin users to non-statin users.

Table S2 Supplementary Table S2 presents statistical analysis of pooled clinical trial data on R/R MM patient response to venetoclax treatment, comparing one prior line of therapy to >1 prior line.

Table S3 Supplementary Table S3 presents statistical analysis of pooled clinical trial data on R/R MM patient response to venetoclax treatment, comparing t11;14 patients to non-t11;14.

Table S4 Supplementary Table S4 presents statistical analysis of pooled clinical trial data on R/R MM patient response to venetoclax treatment, comparing standard risk to high risk patients.

Table S5 Supplementary Table S5 presents multivariate analysis of pooled clinical trial data on R/R MM patients achieving complete response (CR) or better – variables include statin use, t11;14 status, prior lines of therapy, cytogenetic risk.

Table S6 Supplementary Table S6 presents multivariate analysis of pooled clinical trial data on R/R MM patients achieving stringent complete response (sCR) – variables include statin use, t11;14 status, prior lines of therapy, cytogenetic risk.

Table S7 Supplementary Table S7 includes IC50 data for BH3 mimetic drugs in AML cell lines, without or with simvastatin.

Table S8 Supplementary Table S8 provides synergy scores using BLISS and HSA algorithms.

Table S9 Supplementary Table S9 shows the cytogenetic characteristics of the MM patients whose bone marrow cells were studied in Figure 4D.
