# Supplementary material for: Statin-induced Mitochondrial Priming Sensitizes Multiple Myeloma Cells to BCL2 and MCL-1 Inhibitors
Source: Cancer Res Commun. 2023 Dec 8;3(12):2497–509. doi: 10.1158/2767-9764.CRC-23-0350 (PMC10704957; doi:10.1158/2767-9764.CRC-23-0350)
Supplement: Figure S1 — contains additional data from retrospective analysis of venetoclax clinical trials in MM [file crc-23-0350-s01.pdf]

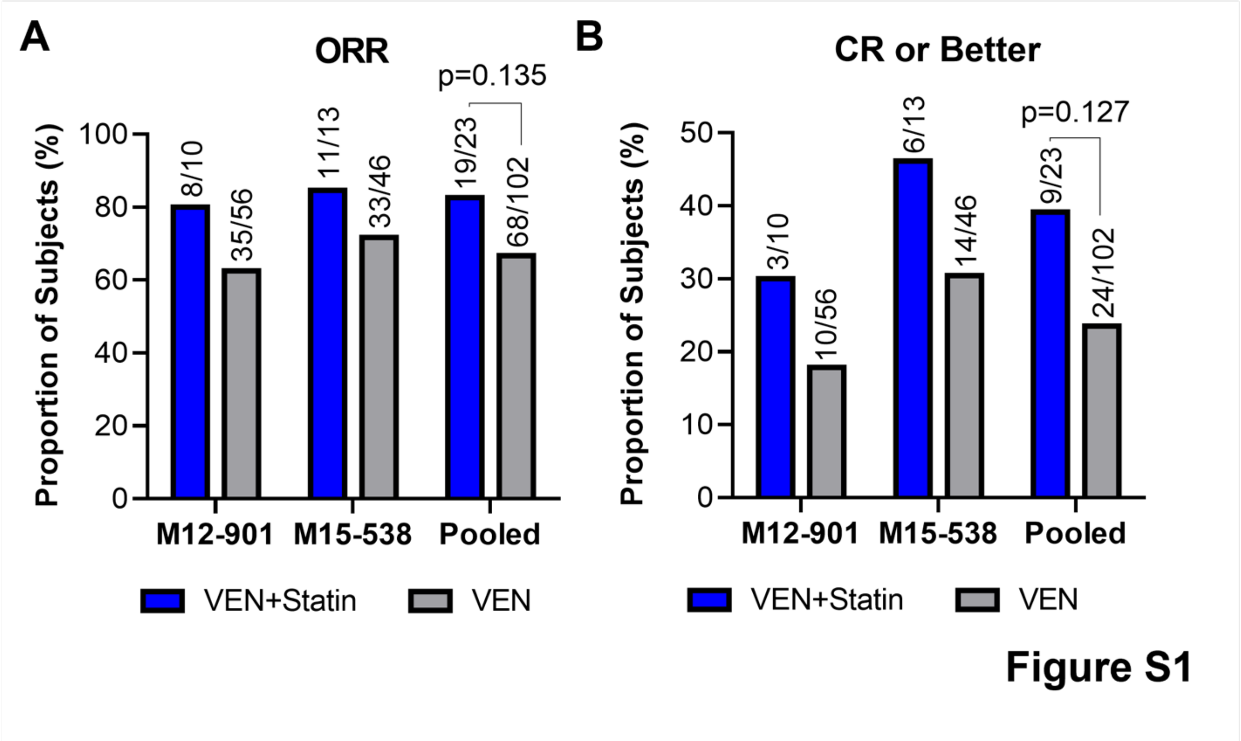

Fig S1: **Statin use trends towards improved responses in a retrospective analysis of venetoclax clinical trials in R/R MM.** A univariate analysis as in Figure 1 was applied to the variables ORR (**A**) or CR or better (**B**). Details of the data analysis are in Table S1.
