# Supplementary material for: Statin-induced Mitochondrial Priming Sensitizes Multiple Myeloma Cells to BCL2 and MCL-1 Inhibitors
Source: Cancer Res Commun. 2023 Dec 8;3(12):2497–509. doi: 10.1158/2767-9764.CRC-23-0350 (PMC10704957; doi:10.1158/2767-9764.CRC-23-0350)
Supplement: Figure S2 — Supplementary Figure 2 contains PFS data from venetoclax clinical trials in MM [file crc-23-0350-s02.pdf]

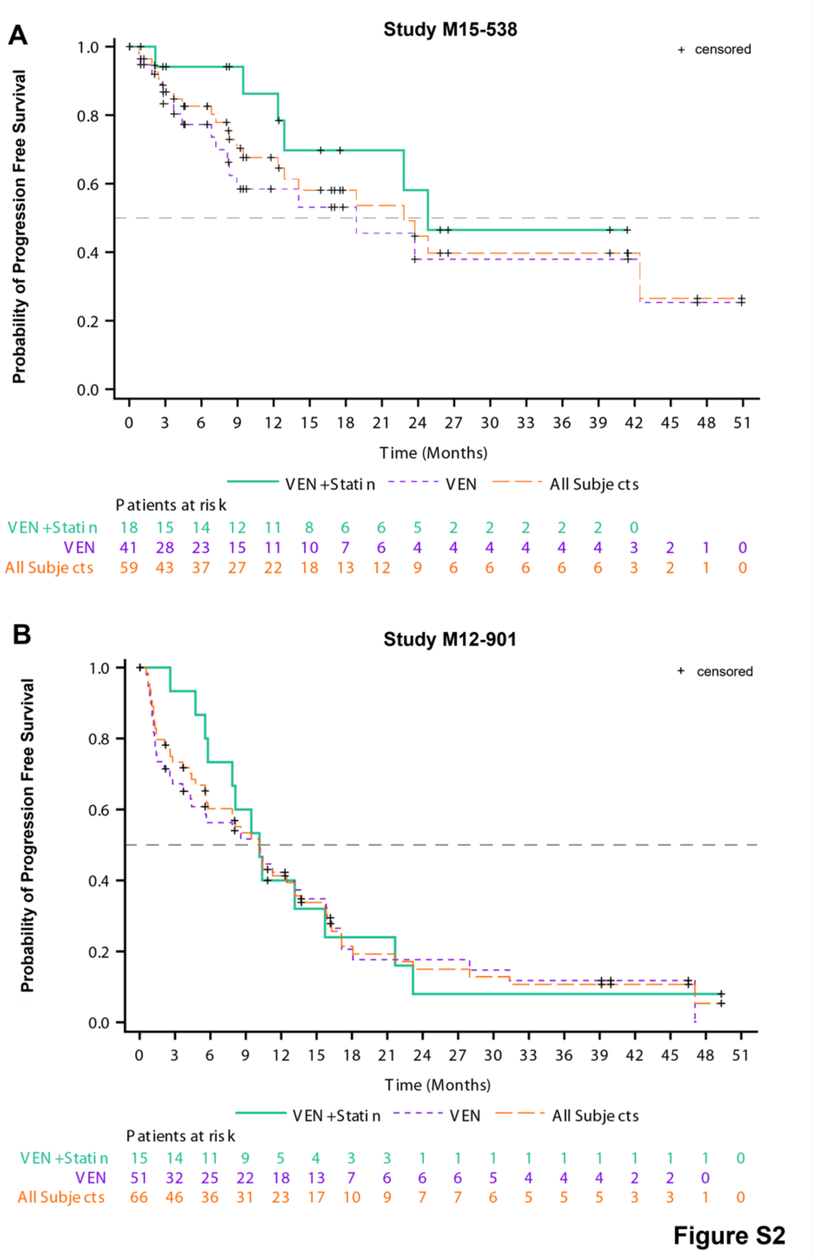

Fig S2: **Statin use trends towards longer PFS in one of the two venetoclax clinical trials.**

VEN = subjects with at least one dose of venetoclax and without use of statin; VEN + Statin = subjects with at least one dose of venetoclax and with use of statin.

A. Trial M15-538 is an ongoing phase II trial of venetoclax/dexamethasone/carfilzomib; the data cutoff date for this PFS analysis was 30 May, 2021.

B. Trial M12-901 is a completed phase 1b trial of venetoclax/dexamethasone/bortezomib.
