# Supplementary material for: Statin-induced Mitochondrial Priming Sensitizes Multiple Myeloma Cells to BCL2 and MCL-1 Inhibitors
Source: Cancer Res Commun. 2023 Dec 8;3(12):2497–509. doi: 10.1158/2767-9764.CRC-23-0350 (PMC10704957; doi:10.1158/2767-9764.CRC-23-0350)
Supplement: Figure S3 — Supplementary Figure 3 contains additional viability assay data and synergy contour plots [file crc-23-0350-s03.pdf]

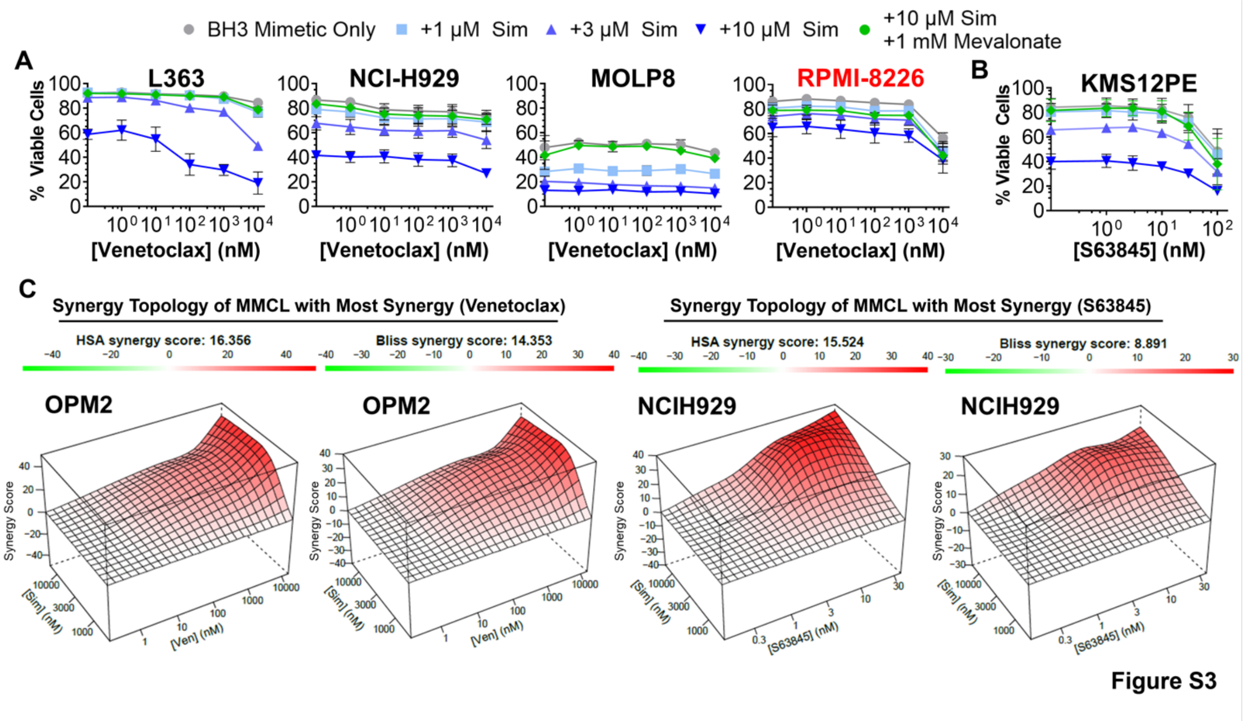

Fig S3: **Additional MMCL sensitivity to BH3 mimetics under increasing simvastatin dose.**

A dose titration of BH3 mimetics was exposed to increasing concentrations of statins. Specificity of statins was assessed by rescue with mevalonate at the highest simvastatin concentration tested.

A. Venetoclax treated combinations with simvastatin.

B. S63845 combinations not pictured in Figure 1A but summarized in 1C. N=3.

C. Exemplary HSA and Bliss synergy topology maps of the MMCL with most synergistic effect observed in venetoclax combinations (OPM2) and S63845 combinations (NCIH929). Remainder of results are summarized in table S8.
