# Supplementary material for: Statin-induced Mitochondrial Priming Sensitizes Multiple Myeloma Cells to BCL2 and MCL-1 Inhibitors
Source: Cancer Res Commun. 2023 Dec 8;3(12):2497–509. doi: 10.1158/2767-9764.CRC-23-0350 (PMC10704957; doi:10.1158/2767-9764.CRC-23-0350)
Supplement: Figure S4 — Supplementary Figure 4 contains BH3 profiling data, and viability assays in cells expressing p53 dominant negative [file crc-23-0350-s04.pdf]

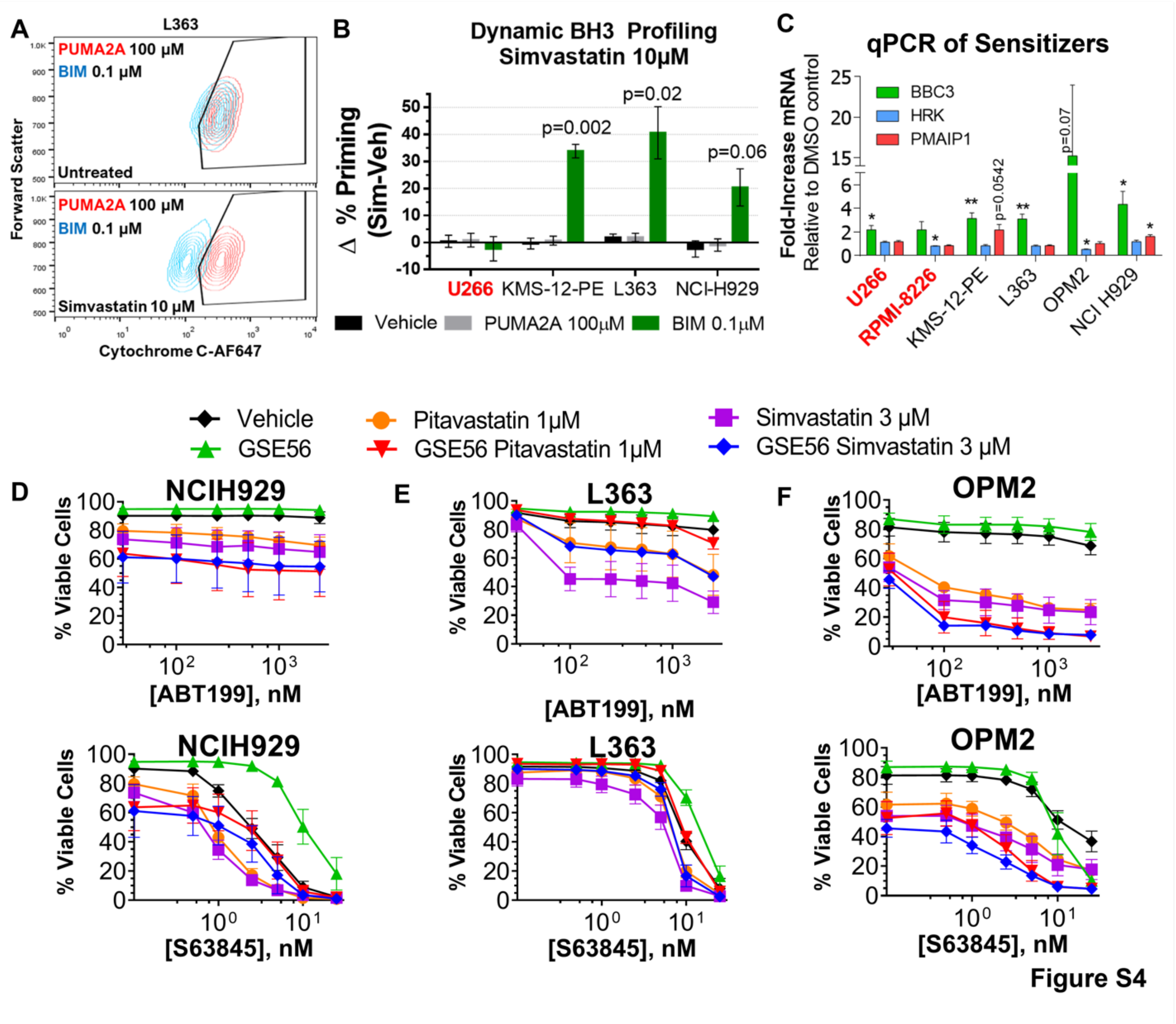

Fig S4: p53 independent PUMA upregulation at 16 hours coincides with sensitization to apoptosis.

A. Exemplary FACS plots depicting cytochrome c release measured by dynamic BH3 profiling with the BIM peptide (blue) and the negative control peptide PUMA2A (red). The percentage of cells that have exited the gate is referred to as % Priming.

B. Change in precent priming caused by simvastatin treatment and probed with the BIM peptide. n=3. Significance determined by one-sample t-test compared to 10% threshold for biological significance. * $p < 0.05$, ** $p < 0.01$, *** $p < 0.001$, ns = not significant.

C. Quantitative PCR results of apoptotic sensitizers. n=4 for U266, KMS-12-PE, and L363. n=3 for OPM2, NCIH929 and RPMI-8226. Significance ($\alpha<0.05$) was determined by one-sample t test of log transformed fold changes against the value of 0.

D - F. Dose response curve of statin/BH3 mimetic combinations in parental or GSE56 expressing (D) NCI-H929 (n=3) (E) OPM2 (n=3), and (F) L363 (n=3) MMCLs.
