# Supplementary material for: Statin-induced Mitochondrial Priming Sensitizes Multiple Myeloma Cells to BCL2 and MCL-1 Inhibitors
Source: Cancer Res Commun. 2023 Dec 8;3(12):2497–509. doi: 10.1158/2767-9764.CRC-23-0350 (PMC10704957; doi:10.1158/2767-9764.CRC-23-0350)
Supplement: Figure S5 — Supplementary Figure 5 contains viability data and PUMA expression data from PBMCs [file crc-23-0350-s05.pdf]

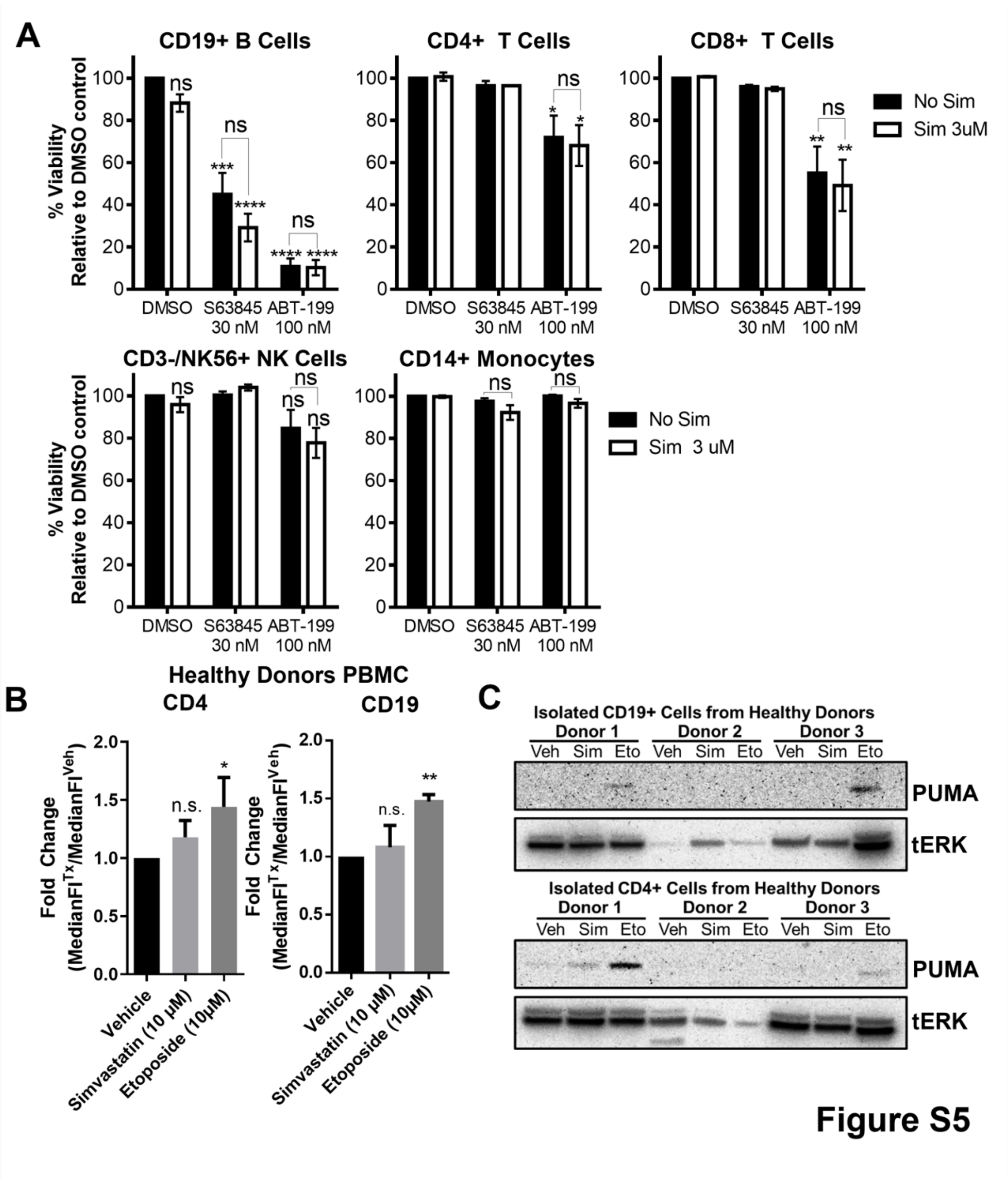

Fig S5: Simvastatin does not promote apoptosis by BH3 Mimetics in Normal PBMC.

A. Healthy donor PBMCs were treated with simvastatin and S63845 or venetoclax and then stained for the indicated cell surface markers and annexin V. Simvastatin at 3 µM did not further sensitize to venetoclax or S63845. Significance determined by two-way ANOVA with Tukey's multiple comparison test.

B. PUMA was measured in CD4 or CD19+ lymphocytes by intracellular staining. PBMCs were treated with simvastatin or etoposide as a positive control. Etoposide induced increase PUMA staining while simvastatin did not. Significance determined one-way ANOVA with Bonferroni's multiple comparison test.

C. Western analysis of isolated lymphocytes from healthy PBMC donors after 16hr treatment. Magnetic beads were used to isolate CD19 and CD4+ cells for western analysis of PUMA. PUMA could only be consistently detected in lymphocytes treated with etoposide.
