# Supplementary material for: Statin-induced Mitochondrial Priming Sensitizes Multiple Myeloma Cells to BCL2 and MCL-1 Inhibitors
Source: Cancer Res Commun. 2023 Dec 8;3(12):2497–509. doi: 10.1158/2767-9764.CRC-23-0350 (PMC10704957; doi:10.1158/2767-9764.CRC-23-0350)
Supplement: Figure S6 — Supplementary Figure 6 contains viability data from cells treated with the GGPP synthase inhibitor TH-Z145 [file crc-23-0350-s06.pdf]

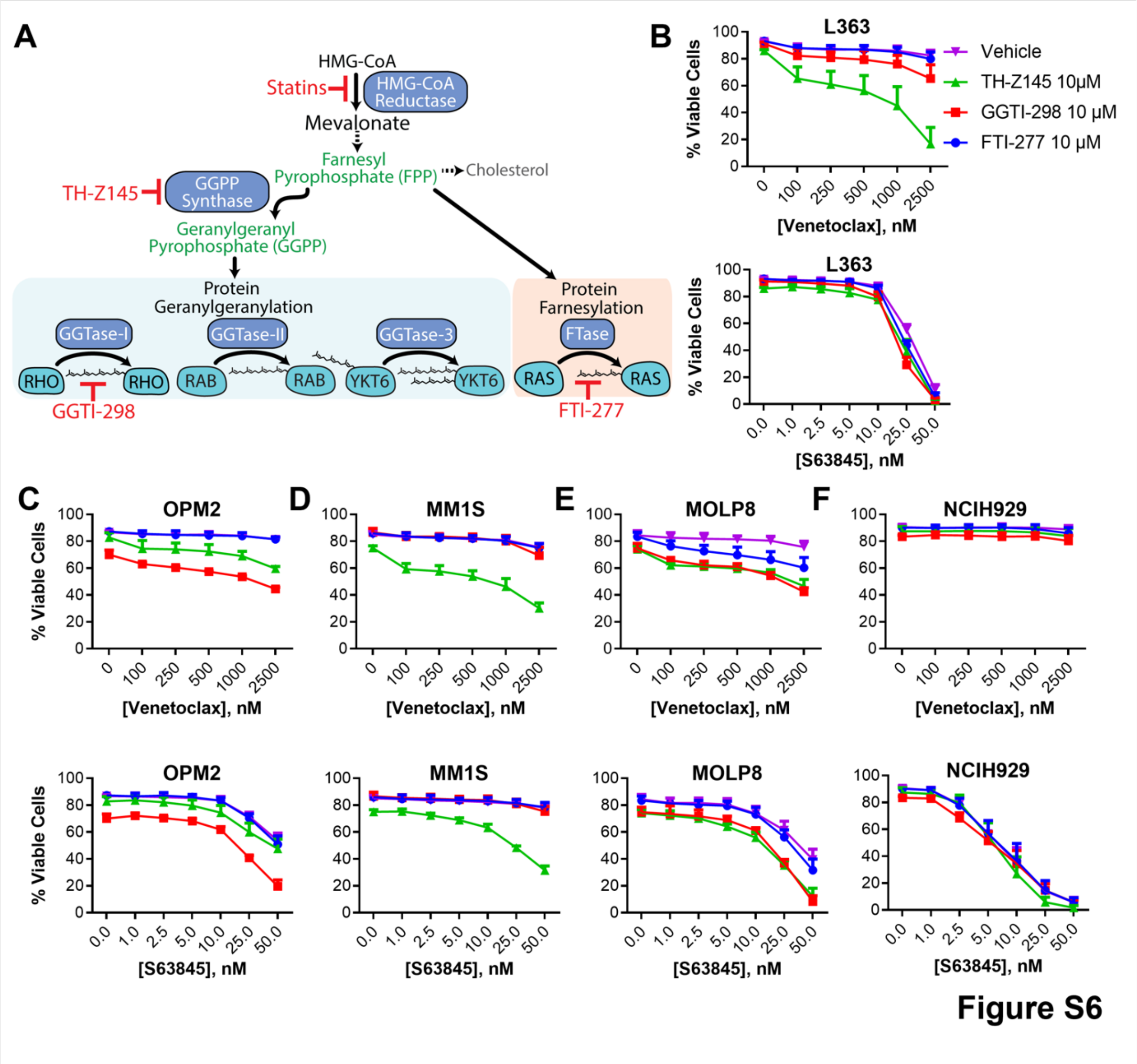

Fig S6: Inhibition of Protein Geranylgeranylation Sensitizes to BH3 Mimetics.

A. Statins impart their effects by depleting FPP and GGPP used for the covalent addition of farnesyl or geranylgeranyl moieties to proteins. FTI-277 is a CAAX-mimetic that inhibits the farnesyltransferase (FTase) enzyme that predominantly acts on Ras family proteins. GGTI-298 is a CAAX-mimetic that inhibits the geranylgeranyltransferase(GGTase)-I enzyme that predominantly acts on Rho/Rac/Rap family proteins. Two additional GGTase enzymes exist with

different substrate binding capacities. GGTase-II modifies RAB proteins whereas the novel GGTase-3 acts on YKT6 and FBXL2 and likely other yet to be described substrates. TH-Z145 is a bisphosphonate-like inhibitor of the GGPP synthase that can specifically deplete GGPP and inhibit all geranylgeranyltransferases.

B-F. Results of 48hr annexin V-PI viability assay using the indicated concentrations of each inhibitor.
