# Supplementary material for: Statin-induced Mitochondrial Priming Sensitizes Multiple Myeloma Cells to BCL2 and MCL-1 Inhibitors
Source: Cancer Res Commun. 2023 Dec 8;3(12):2497–509. doi: 10.1158/2767-9764.CRC-23-0350 (PMC10704957; doi:10.1158/2767-9764.CRC-23-0350)
Supplement: Figure S7 — Supplementary Figure 7 contains data comparing pitavastatin with simavastatin in western blots (panel A) and BH3 profiling data with pitavastatin (panel B). [file crc-23-0350-s07.pdf]

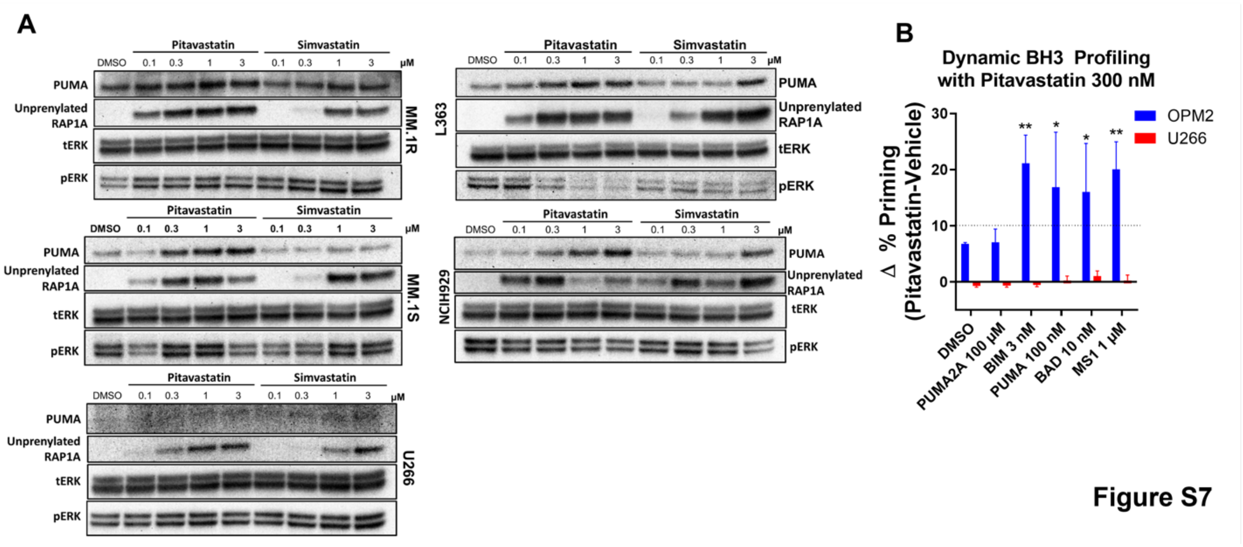

Fig S7: Pitavastatin activity on biomarkers of prenylation and cell death.

A. Pitavastatin increases PUMA, blocks prenylation, and affects phosphorylated ERK at concentrations lower than simvastatin in several MMCLs

B. 300 nM Pitavastatin sensitizes to apoptosis as measured by BH3 profiling in OPM2. Significance determined by student’s t-test against untreated samples.
