# Supplementary material for: Statin-induced Mitochondrial Priming Sensitizes Multiple Myeloma Cells to BCL2 and MCL-1 Inhibitors
Source: Cancer Res Commun. 2023 Dec 8;3(12):2497–509. doi: 10.1158/2767-9764.CRC-23-0350 (PMC10704957; doi:10.1158/2767-9764.CRC-23-0350)
Supplement: Figure S8 — Supplementary Figure 8 contains data on GILZ mRNA expression in MM1S and MM1R cells [file crc-23-0350-s08.pdf]

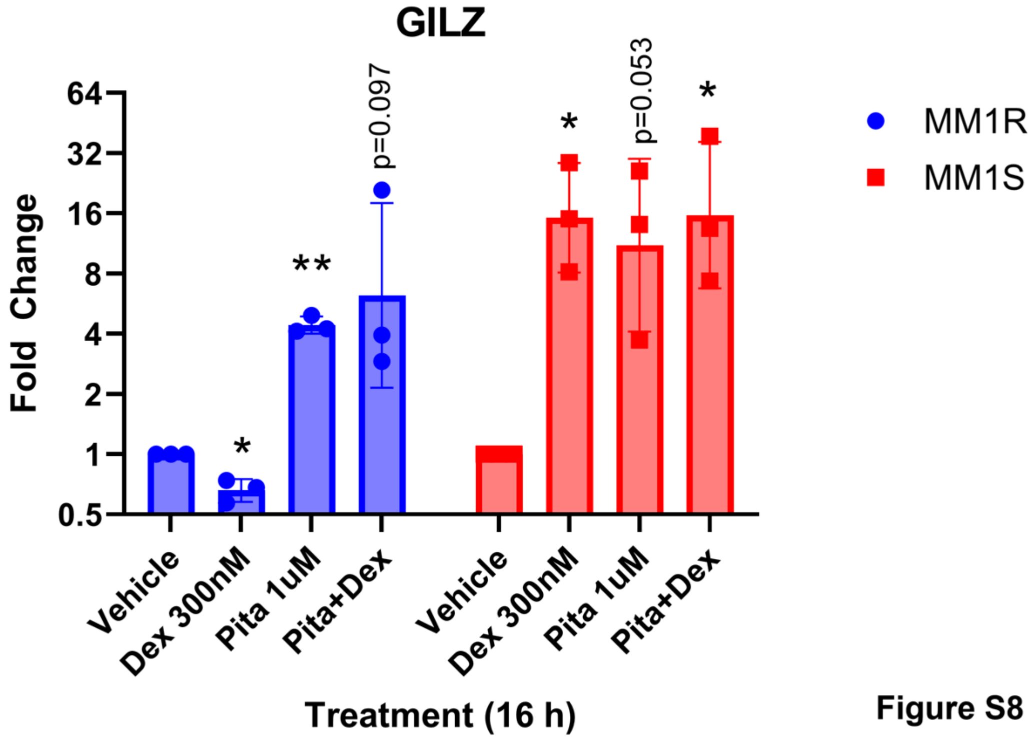

Fig S8: Pitavastatin and Dexamethasone Upregulate GILZ. Statins increase GILZ transcript expression in a dexamethasone resistance model as measured by qPCR. Significance ($p<0.05$) determined by one-sample t test of log transformed fold changes against the value of 0.
