# Supplementary material for: Statin-induced Mitochondrial Priming Sensitizes Multiple Myeloma Cells to BCL2 and MCL-1 Inhibitors
Source: Cancer Res Commun. 2023 Dec 8;3(12):2497–509. doi: 10.1158/2767-9764.CRC-23-0350 (PMC10704957; doi:10.1158/2767-9764.CRC-23-0350)
Supplement: Figure S9 — Supplementary Figure 9 contains data on L363 cells with PUMA (BBC3) knockout or inducible knockdown. [file crc-23-0350-s09.pdf]

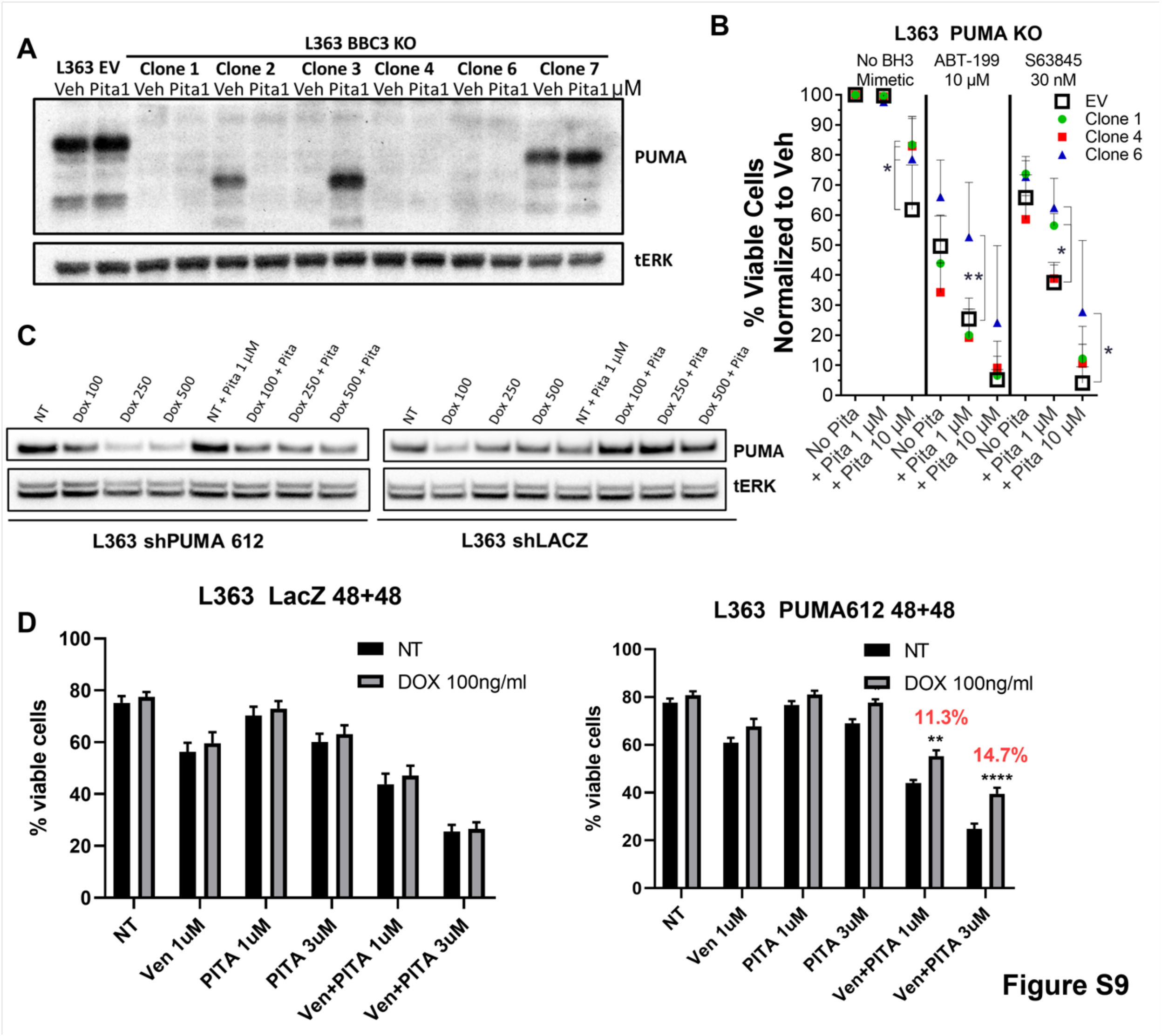

Fig S9: PUMA upregulation partially explains statin-mediated apoptotic sensitization.

A. We used CRISPR-cas9 to generate knockouts of PUMA and confirmed PUMA knockout at the protein level in single cell L363 clones.

B. 48hr Annexin V/PI viability assays were used to assess the effect of PUMA knockout on statin-mediated sensitization of apoptosis in three single cell clones compared to a pool expressing a non-targeting empty vector. Significance was assessed by one-way ANOVA with Tukey's post-test correction for multiple comparisons.

C. Knockdown of PUMA with a doxycycline inducible shRNA was confirmed at the protein level at 100 ng/mL doxycycline for 96 hours.

D. Doxycycline inducible shRNA-mediated knockdown of PUMA from pitavastatin partially attenuated sensitization to venetoclax whereas the shLACZ did not have any effect. Significance determined by student's t test.
