# Supplementary material for: Statin-induced Mitochondrial Priming Sensitizes Multiple Myeloma Cells to BCL2 and MCL-1 Inhibitors
Source: Cancer Res Commun. 2023 Dec 8;3(12):2497–509. doi: 10.1158/2767-9764.CRC-23-0350 (PMC10704957; doi:10.1158/2767-9764.CRC-23-0350)
Supplement: Figure S10 — Supplementary Figure 10 contains additional data on ISR-dependent changes in protein synthesis rates and viability. [file crc-23-0350-s10.pdf]

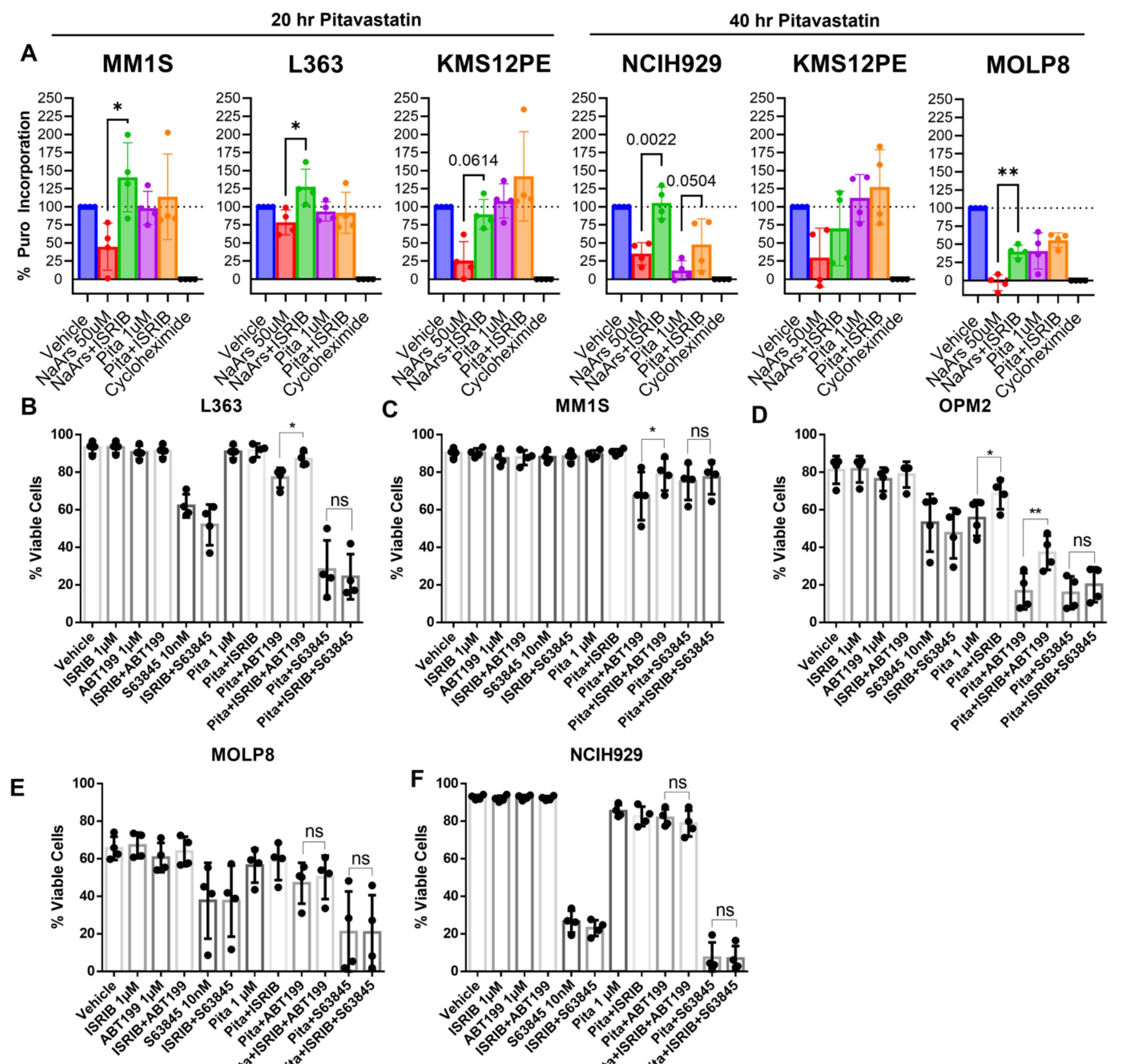


**Figure S10**

**Fig S10: ISRIB Rescues Statin-Mediated Sensitization to Venetoclax, but Not S63845.**

**A.** Pitavastatin-induced reduction of puromycin incorporation assay is time dependent and cell line dependent. MM1S, L363 and MOLP-8 do not experience reductions in translation rate at 20hrs, but do at 40hrs. NCI-H929 experiences a reduction in puromycin incorporation at 20 hrs and at 40hrs ISRIB (1 μM) can only partially rescue the reduction in translation rate.

KMS12PE does not experience ISR at 20 or 40hrs consistent with western analysis. These experiments were performed and analyzed as described in Figure 5B.

**B-E**. Cells treated with statin/BH3 mimetic combinations plus or minus ISRIB for 48 hours. The presence of ISRIB partially rescues from venetoclax mediated killing in L363, MM1S, and OPM2 cells, but not from S63845 mediated killing. MOLP8 and NCI-H929, which are not sensitive to venetoclax, are not rescued by ISRIB. Significance is determined by multiple student t tests corrected by Bonferroni.
