# Supplementary material for: Statin-induced Mitochondrial Priming Sensitizes Multiple Myeloma Cells to BCL2 and MCL-1 Inhibitors
Source: Cancer Res Commun. 2023 Dec 8;3(12):2497–509. doi: 10.1158/2767-9764.CRC-23-0350 (PMC10704957; doi:10.1158/2767-9764.CRC-23-0350)
Supplement: Figure S11 — Supplementary Figure 11 presents data showing that pitavastatin does not activate ISR in AML cell lines. [file crc-23-0350-s11.pdf]

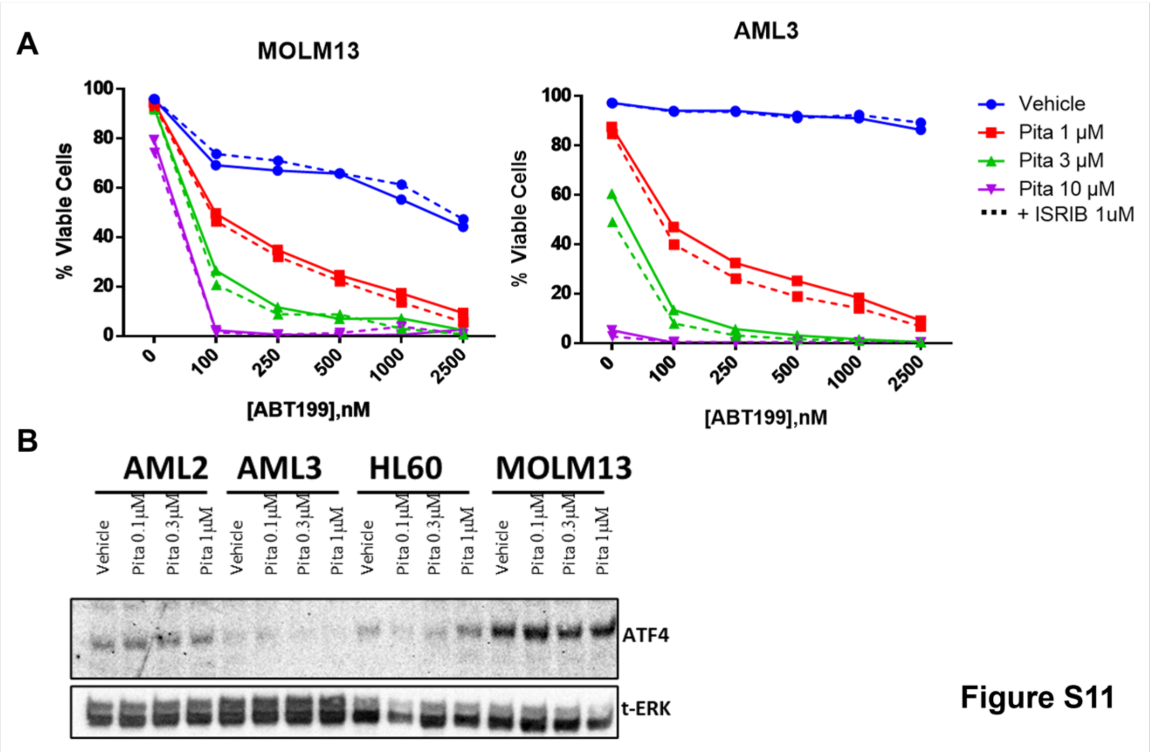

Fig S11: ISRIB does not rescue from statin-mediated apoptotic sensitization in AML Cell Lines.

A. The statin sensitive AML cell lines, MOLM13 and OCI-AML3 (AML3) are not rescued with ISRIB, n = 1.

B. AML cell lines do not upregulate ATF4 in response to pitavastatin treatment. Similar results were observed in a second experiment using AML3 cells.
