# Supplementary material for: Statin-induced Mitochondrial Priming Sensitizes Multiple Myeloma Cells to BCL2 and MCL-1 Inhibitors
Source: Cancer Res Commun. 2023 Dec 8;3(12):2497–509. doi: 10.1158/2767-9764.CRC-23-0350 (PMC10704957; doi:10.1158/2767-9764.CRC-23-0350)
Supplement: Figure S12 — Supplementary Figure 12 contains additional data on the role of PERK in statin-induced ISR in MM cell lines. [file crc-23-0350-s12.pdf]

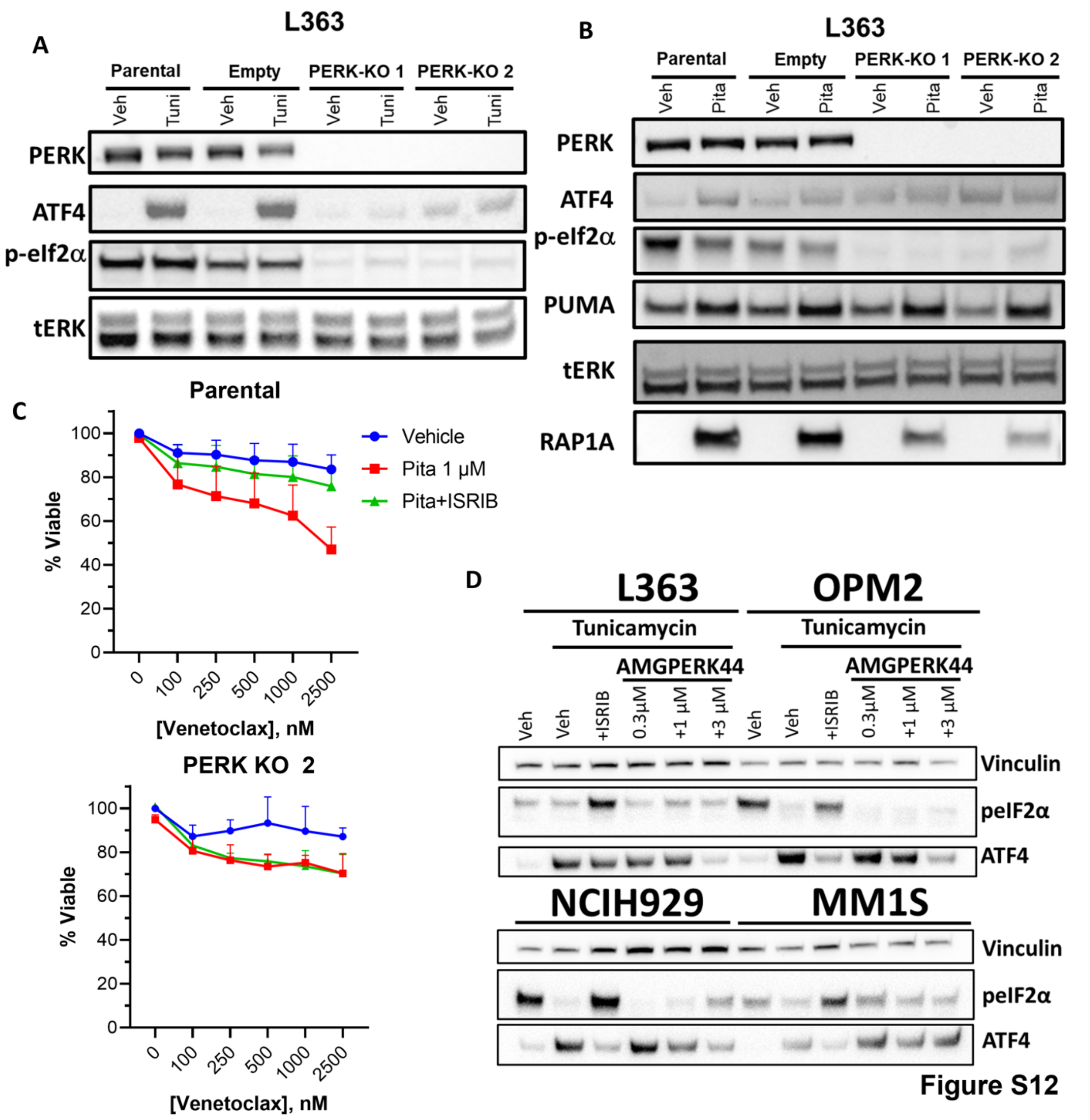

Fig S12: ISR induced by pitavastatin is due to PERK in L363, but other kinases may play a role in different MMCLs. PERK knockout (KO) was achieved in two clones of L363

A. Knockout was confirmed blotting for PERK and by 16-hour treatment with tunicamycin. KO clones had reduced phosphorylated eIF2α and were not able to upregulate ATF4 in response to tunicamycin 5 μg/mL.

B. Cells were treated with pitavastatin 1 μM for 40hrs to induce ATF4 upregulation. PERK knockout blocked ATF4 increases in the two knockout clones.

C. ISRIB can no longer rescue from the pitavastatin-mediated venetoclax sensitization in a 40hr Annexin V-PI assay of the PERK-KO clone 2. Mean +/- SD, n=2.

D. An effective concentration of the PERK inhibitor AMG-PERK44 was selected by dose titration under 5 μg/mL Tunicamycin to activate PERK in L363, OPM2, NCIH929, and MM1S. 3 μM AMG-PERK44 was sufficient to block tunicamycin-mediated ATF4 induction in L363, OPM2, and NCIH929.
