# Supplementary material for: Statin-induced Mitochondrial Priming Sensitizes Multiple Myeloma Cells to BCL2 and MCL-1 Inhibitors
Source: Cancer Res Commun. 2023 Dec 8;3(12):2497–509. doi: 10.1158/2767-9764.CRC-23-0350 (PMC10704957; doi:10.1158/2767-9764.CRC-23-0350)
Supplement: Figure S13 — Supplementary Figure 13 presents data on Cas9-mediated NOXA and PUMA deletion in KMS18 cells (panel A) and viability assays in cells with PUMA knockdown and ISRIB treatment (panel B). [file crc-23-0350-s13.pdf]

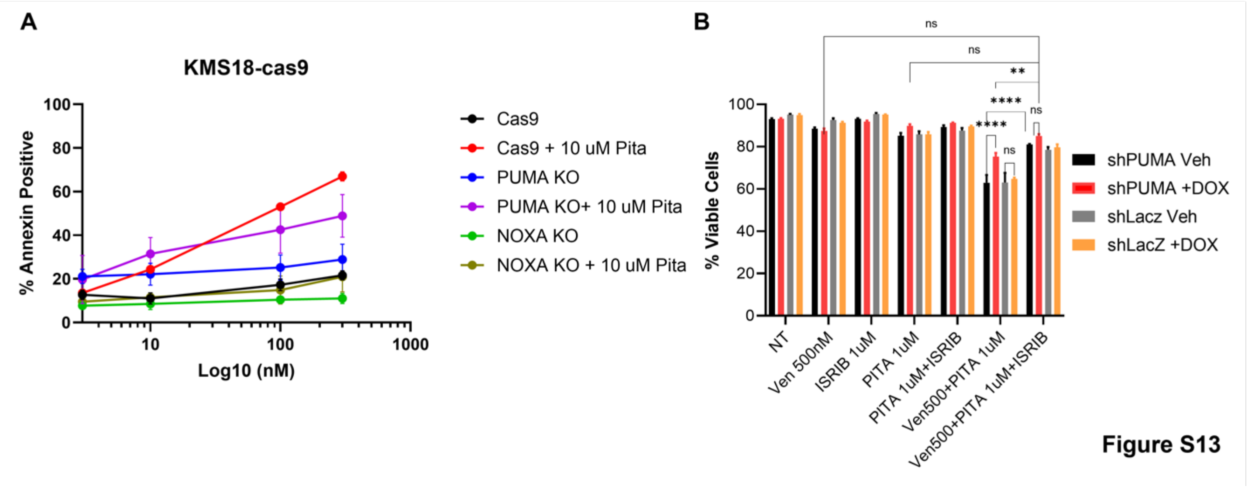

Fig S13: Additional data on PUMA and NOXA

A. PUMA and NOXA Contribute to Statin-mediated Apoptotic Sensitization in KMS18. KMS18 cells with inducible cas9 were used to generate NOXA and PUMA knockouts. NOXA knockout cell lines lose sensitivity to venetoclax while PUMA knockout cell lines partially lose apoptotic sensitization induced by statins. Mean +/-SEM, n = 3.

B. A doxycycline inducible shRNA against *BBC3* (PUMA) partially rescues from pitavastatin-mediated venetoclax sensitization in L363 after 100 ng/mL doxycycline treatment. ISRIB also partially rescues from sensitization. PUMA knockdown and ISRIB exposure rescues completely from pitavastatin-mediated venetoclax sensitization. Mean +/- SD, n=4, significance determined by two-way ANOVA and multiple comparisons were corrected by Tukey’s multiple comparison test.
