# Supplementary material for: Statin-induced Mitochondrial Priming Sensitizes Multiple Myeloma Cells to BCL2 and MCL-1 Inhibitors
Source: Cancer Res Commun. 2023 Dec 8;3(12):2497–509. doi: 10.1158/2767-9764.CRC-23-0350 (PMC10704957; doi:10.1158/2767-9764.CRC-23-0350)
Supplement: Table S1 — Supplementary Table S1 presents statistical analysis of pooled clinical trial data on R/R MM patient response to venetoclax treatment, comparing statin users to non-statin users. [file crc-23-0350-s14.pdf]

Table S1: Univariate analysis of pooled clinical trial data on R/R MM patient response to venetoclax treatment grouped by statin use. "VEN + Statin" includes patients with at least one dose of venetoclax and with use of Statin. VEN + Statin patients received at least 1 statin medication given before the first response date. "VEN" includes patients with at least one dose of venetoclax and without use of Statin. VEN may include patients treated with only VEN or patients that started a statin after the first response date. The 95% CI for rate is computed using clopper-pearson method. * P-value <= 0.05; ** P-value <= 0.01; *** P-value < 0.001.

	VEN + Statin (N=23)	VEN (N=102)	All Subjects (N=125)	Two-sided P-value
Best Overall Response	n (%) [95% CI of Rate]	n (%) [95% CI of Rate]	n (%) [95% CI of Rate]	VEN + Statin vs VEN
Stringent Complete Response (sCR)	7 (30.4) [13.2, 52.9]	6 (5.9) [2.2, 12.4]	13 (10.4) [5.7, 17.1]	<0.001***
Complete Response (CR)	2 (8.7) [1.1, 28.0]	18 (17.6) [10.8, 26.4]	20 (16.0) [10.1, 23.6]	0.292
Very Good Partial Response (VGPR)	5 (21.7) [7.5, 43.7]	26 (25.5) [17.4, 35.1]	31 (24.8) [17.5, 33.3]	0.708
Partial Response (PR)	5 (21.7) [7.5, 43.7]	18 (17.6) [10.8, 26.4]	23 (18.4) [12.0, 26.3]	0.649
Overall Response (sCR+CR+VGPR+PR)	19 (82.6) [61.2, 95.0]	68 (66.7) [56.6, 75.7]	87 (69.6) [60.7, 77.5]	0.135
Complete Response or Better (sCR+CR)	9 (39.1) [19.7, 61.5]	24 (23.5) [15.7, 33.0]	33 (26.4) [18.9, 35.0]	0.127
