# Supplementary material for: Statin-induced Mitochondrial Priming Sensitizes Multiple Myeloma Cells to BCL2 and MCL-1 Inhibitors
Source: Cancer Res Commun. 2023 Dec 8;3(12):2497–509. doi: 10.1158/2767-9764.CRC-23-0350 (PMC10704957; doi:10.1158/2767-9764.CRC-23-0350)
Supplement: Table S2 — Supplementary Table S2 presents statistical analysis of pooled clinical trial data on R/R MM patient response to venetoclax treatment, comparing one prior line of therapy to >1 prior line. [file crc-23-0350-s15.pdf]

Table S2: Univariate analysis of pooled clinical trial data on R/R MM patient response to venetoclax treatment grouped by prior lines of therapy =1 or >1. "VEN + 1 therapy" includes patients with at least one dose of venetoclax and with 1 line of prior therapy. "VEN + > 1 therapy" includes patients with at least one dose of Venetoclax with > 1 lines of prior therapy. P-value is based on CMH test. The 95% CI for rate is computed using clopper-pearson method. * P-value <= 0.05; ** P-value <= 0.01; *** P-value < 0.001.

	VEN + 1 Therapy (N=49)	VEN + >1 Therapy (N=76)	All Subjects (N=125)	Two-sided P-value
Best Overall Response	n (%) [95% CI of Rate]	n (%) [95% CI of Rate]	n (%) [95% CI of Rate]	VEN + 1 Therapy vs VEN + >1 Therapy
Stringent Complete Response (sCR)	5 (10.2) [3.4, 22.2]	8 (10.5) [4.7, 19.7]	13 (10.4) [5.7, 17.1]	0.954
Complete Response (CR)	12 (24.5) [13.3, 38.9]	8 (10.5) [4.7, 19.7]	20 (16.0) [10.1, 23.6]	0.038*
Very Good Partial Response (VGPR)	12 (24.5) [13.3, 38.9]	19 (25.0) [15.8, 36.3]	31 (24.8) [17.5, 33.3]	0.949
Partial Response (PR)	8 (16.3) [7.3, 29.7]	15 (19.7) [11.5, 30.5]	23 (18.4) [12.0, 26.3]	0.632
Overall Response (sCR+CR+VGPR+PR)	37 (75.5) [61.1, 86.7]	50 (65.8) [54.0, 76.3]	87 (69.6) [60.7, 77.5]	0.251
Complete Response or Better (sCR+CR)	17 (34.7) [21.7, 49.6]	16 (21.1) [12.5, 31.9]	33 (26.4) [18.9, 35.0]	0.092
