# Supplementary material for: Statin-induced Mitochondrial Priming Sensitizes Multiple Myeloma Cells to BCL2 and MCL-1 Inhibitors
Source: Cancer Res Commun. 2023 Dec 8;3(12):2497–509. doi: 10.1158/2767-9764.CRC-23-0350 (PMC10704957; doi:10.1158/2767-9764.CRC-23-0350)
Supplement: Table S3 — Supplementary Table S3 presents statistical analysis of pooled clinical trial data on R/R MM patient response to venetoclax treatment, comparing t11;14 patients to non-t11;14. [file crc-23-0350-s16.pdf]

Table S3: Univariate analysis of pooled clinical trial data on R/R MM patient response to venetoclax treatment grouped by t(11;14) status. "VEN + T1114+" includes patients with at least one dose of venetoclax and with the t(11;14) translocation. "VEN + T1114-" includes patients with at least one dose of venetoclax without the t(11;14) translocation. P-value is based on CMH test. The 95% CI for rate is computed using clopper-pearson method. * P-value <= 0.05; ** P-value <= 0.01; *** P-value < 0.001.

	VEN + T1114+ (N=32)	VEN + T1114- (N=87)	All Subjects (N=119)	Two-sided P-value
Best Overall Response	n (%) [95% CI of Rate]	n (%) [95% CI of Rate]	n (%) [95% CI of Rate]	VEN + T1114+ vs VEN + T1114-
Stringent Complete Response (sCR)	3 (9.4) [2.0, 25.0]	10 (11.5) [5.7, 20.1]	13 (10.9) [5.9, 18.0]	0.744
Complete Response (CR)	6 (18.8) [7.2, 36.4]	11 (12.6) [6.5, 21.5]	17 (14.3) [8.5, 21.9]	0.401
Very Good Partial Response (VGPR)	8 (25.0) [11.5, 43.4]	22 (25.3) [16.6, 35.7]	30 (25.2) [17.7, 34.0]	0.975
Partial Response (PR)	7 (21.9) [9.3, 40.0]	14 (16.1) [9.1, 25.5]	21 (17.6) [11.3, 25.7]	0.465
Overall Response (sCR+CR+VGPR+PR)	24 (75.0) [56.6, 88.5]	57 (65.5) [54.6, 75.4]	81 (68.1) [58.9, 76.3]	0.327
Complete Response or Better (sCR+CR)	9 (28.1) [13.7, 46.7]	21 (24.1) [15.6, 34.5]	30 (25.2) [17.7, 34.0]	0.658
