# Supplementary material for: Statin-induced Mitochondrial Priming Sensitizes Multiple Myeloma Cells to BCL2 and MCL-1 Inhibitors
Source: Cancer Res Commun. 2023 Dec 8;3(12):2497–509. doi: 10.1158/2767-9764.CRC-23-0350 (PMC10704957; doi:10.1158/2767-9764.CRC-23-0350)
Supplement: Table S4 — Supplementary Table S4 presents statistical analysis of pooled clinical trial data on R/R MM patient response to venetoclax treatment, comparing standard risk to high risk patients. [file crc-23-0350-s17.pdf]

Table S4: Univariate analysis of pooled clinical trial data on R/R MM patient response to venetoclax treatment grouped by cytogenetic risk status according to IMWG criteria. "VEN + RiskS" includes patients with at least one dose of venetoclax and with standard cytogenetics risk. "VEN + RiskH" includes patients with at least one dose of venetoclax with high cytogenetics risk. P-value is based on CMH test. The 95% CI for rate is computed using clopper-pearson method. * P-value <= 0.05; ** P-value <= 0.01; *** P-value < 0.001.

	VEN + RiskS (N=78)	VEN + RiskH (N=35)	All Subjects (N=113)	Two-sided P-value
Best Overall Response	n (%) [95% CI of Rate]	n (%) [95% CI of Rate]	n (%) [95% CI of Rate]	VEN + RiskS vs VEN + RiskH
Stringent Complete Response (sCR)	11 (14.1) [7.3, 23.8]	0 [0.0, 10.0]	11 (9.7) [5.0, 16.8]	0.020*
Complete Response (CR)	8 (10.3) [4.5, 19.2]	8 (22.9) [10.4, 40.1]	16 (14.2) [8.3, 22.0]	0.077
Very Good Partial Response (VGPR)	19 (24.4) [15.3, 35.4]	11 (31.4) [16.9, 49.3]	30 (26.5) [18.7, 35.7]	0.433
Partial Response (PR)	17 (21.8) [13.2, 32.6]	4 (11.4) [3.2, 26.7]	21 (18.6) [11.9, 27.0]	0.192
Overall Response (sCR+CR+VGPR+PR)	55 (70.5) [59.1, 80.3]	23 (65.7) [47.8, 80.9]	78 (69.0) [59.6, 77.4]	0.612
Complete Response or Better (sCR+CR)	19 (24.4) [15.3, 35.4]	8 (22.9) [10.4, 40.1]	27 (23.9) [16.4, 32.8]	0.863
