# Supplementary material for: Statin-induced Mitochondrial Priming Sensitizes Multiple Myeloma Cells to BCL2 and MCL-1 Inhibitors
Source: Cancer Res Commun. 2023 Dec 8;3(12):2497–509. doi: 10.1158/2767-9764.CRC-23-0350 (PMC10704957; doi:10.1158/2767-9764.CRC-23-0350)
Supplement: Table S5 — Supplementary Table S5 presents multivariate analysis of pooled clinical trial data on R/R MM patients achieving complete response (CR) or better – variables include statin use, t11;14 status, prior lines of therapy, cytogenetic risk. [file crc-23-0350-s18.pdf]

Table S5: Multivariate analysis of pooled clinical trial data on R/R MM patients achieving complete response (CR) or better. Three baseline characteristics were considered in addition to statin usage as independent variables in a multivariate analysis to evaluate their association with achieving a CR or better, including t(11;14 status), prior lines of therapy, and cytogenetics risk.

Coefficients	Estimate	Std.Error	P-value	Odds Ratio	Lower 95% CI	Upper 95% CI
Intercept	-0.915	0.512	0.074			
T(11;14) Status (Positive vs Negative)	0.539	0.519	0.299	1.714	0.62	4.742
Prior Lines Therapy (> 1 vs 1)	-0.847	0.467	0.07	0.429	0.172	1.07
Cytogenetics Risk (Standard vs High)	-0.156	0.504	0.757	0.856	0.319	2.297
Statin Usage (Yes vs No)	1.02	0.544	0.061	2.773	0.954	8.055
