# Supplementary material for: Statin-induced Mitochondrial Priming Sensitizes Multiple Myeloma Cells to BCL2 and MCL-1 Inhibitors
Source: Cancer Res Commun. 2023 Dec 8;3(12):2497–509. doi: 10.1158/2767-9764.CRC-23-0350 (PMC10704957; doi:10.1158/2767-9764.CRC-23-0350)
Supplement: Table S6 — Supplementary Table S6 presents multivariate analysis of pooled clinical trial data on R/R MM patients achieving stringent complete response (sCR) – variables include statin use, t11;14 status, prior lines of therapy, cytogenetic risk. [file crc-23-0350-s19.pdf]

**Table S6: Multivariate analysis of pooled clinical trial data on R/R MM patients achieving stringent complete response.** Two baseline characteristics were considered in addition to statin usage as independent variables in a multivariate analysis to evaluate their association with achieving a CR or better, including t(11;14 status) and prior lines of therapy. Cytogenetic risk was not considered as zero patients with high cytogenetic risk achieved stringent complete response. Statin usage is statistically significantly associated with achieving stringent complete response when accounting for t(11;14) status and prior lines of therapy. * P-value <= 0.05; ** P-value <= 0.01; *** P-value < 0.001.

| Coefficients | Estimate | Std.Error | P-value | Odds Ratio | Lower 95% CI | Upper 95% CI |
|---|---|---|---|---|---|---|
| Intercept | -2.673 | 0.587 | <0.001*** | | | |
| T(11;14) Status (Positive vs Negative) | 0.172 | 0.751 | 0.819 | 1.187 | 0.273 | 5.171 |
| Prior Lines Therapy (> 1 vs 1) | -0.172 | 0.649 | 0.791 | 0.842 | 0.236 | 3.004 |
| Statin Usage (Yes vs No) | 2.004 | 0.644 | 0.002** | 7.415 | 2.097 | 26.221 |
