# Supplementary material for: Statin-induced Mitochondrial Priming Sensitizes Multiple Myeloma Cells to BCL2 and MCL-1 Inhibitors
Source: Cancer Res Commun. 2023 Dec 8;3(12):2497–509. doi: 10.1158/2767-9764.CRC-23-0350 (PMC10704957; doi:10.1158/2767-9764.CRC-23-0350)
Supplement: Table S7 — Supplementary Table S7 includes IC50 data for BH3 mimetic drugs in AML cell lines, without or with simvastatin. [file crc-23-0350-s20.pdf]

Table S7: IC50 values for venetoclax and S63845 in MM cells without or with simvastatin. Viability is normalized to the baseline viability of vehicle or simvastatin alone to determine the IC50 of the BH3 mimetic in the respective condition from a variable slope hill model. MMCLs are ordered by most synergy. Significance is determined by extra sum-of-squares F test with an $\alpha = 0.0167$ for 3 multiple comparisons. P values are adjusted for multiple comparisons. * $p < 0.05$, ** $p < 0.01$, *** $p < 0.001$, **** $p < 0.0001$.

MMCL	[Sim] in µM	BH3 Mimetic	BH3 Mimetic IC50 with Veh in nM	BH3 Mimetic IC50 with Sim in nM	P adj
OPM2	3	VEN	>10 µM	3838	0.0009 ***
OPM2	1	VEN	>10 µM	8505	0.13
L363	10	VEN	>10 µM	894.20	< 0.00010 ****
L363	3	VEN	>10 µM	>10 µM	n.s.
KMS12PE	10	VEN	7.37	2.56	< 0.00010 ****
KMS12PE	3	VEN	7.37	3.10	< 0.00010 ****
RPMI8226	10	VEN	>10 µM	>10 µM	n.s.
RPMI8226	3	VEN	>10 µM	>10 µM	n.s.
NCIH929	10	VEN	>10 µM	>10 µM	n.s.
NCIH929	3	VEN	>10 µM	>10 µM	n.s.
U266	10	VEN	>10 µM	>10 µM	n.s.
U266	3	VEN	>10 µM	>10 µM	n.s.
MOLP8	10	VEN	Cannot Fit Hill	Cannot Fit Hill	N/A
MOLP8	3	VEN	Cannot Fit Hill	Cannot Fit Hill	N/A
NCIH929	10	S63845	53.22	8.69	< 0.00010 ****
NCIH929	3	S63845	53.22	16.47	< 0.00010 ****
OPM2	3	S63845	28.50	12.26	< 0.00010 ****
OPM2	1	S63845	28.50	17.81	< 0.00010 ****
L363	10	S63845	25.94	4.72	< 0.00010 ****
L363	3	S63845	25.94	14.21	< 0.00010 ****
MOLP8	10	S63845	12.92	3.59	0.00090 ***
MOLP8	3	S63845	12.92	4.12	0.00090 ***
RPMI8226	10	S63845	93.89	74.64	0.077
RPMI8226	3	S63845	93.89	85.95	0.98
KMS12PE	10	S63845	117.60	75.47	0.016 *
KMS12PE	3	S63845	117.60	93.42	0.41
U266	10	S63845	>10 µM	>10 µM	n.s.
U266	3	S63845	>10 µM	>10 µM	n.s.
