# Supplementary material for: Statin-induced Mitochondrial Priming Sensitizes Multiple Myeloma Cells to BCL2 and MCL-1 Inhibitors
Source: Cancer Res Commun. 2023 Dec 8;3(12):2497–509. doi: 10.1158/2767-9764.CRC-23-0350 (PMC10704957; doi:10.1158/2767-9764.CRC-23-0350)
Supplement: Table S8 — Supplementary Table S8 provides synergy scores using BLISS and HSA algorithms. [file crc-23-0350-s21.pdf]

Table S8: Calculation and Visualization of synergy scores for Drug Combinations using BLISS and HSA algorithms. Synergy is determined by SynergyFinder 3.0 web-application. The "synergy score" is the average of synergy scores over all dose combination measurements for the given synergy model (Bliss: Bliss independence model; HSA: highest single agent) whereas "most synergistic area" describes the average of the most synergistic 3 by 3 dose area in a combination matrix. The antagonism threshold is generally defined as a synergy score less than -10, the additive threshold is defined between -10 and 10, and the synergistic threshold is defined as greater than 10. MMCLs are ordered by the average of the most synergistic area in the Bliss and HSA method.

MMCL	Combination	Synergy Score (Bliss)	Most Synergistic Area (Bliss)	Synergy Score (HSA)	Most Synergistic Area (HSA)
OPM2	Ven + Sim	14.35	22.22	16.36	26.43
L363	Ven + Sim	8.55	17.18	9.65	18.86
KMS12PE	Ven + Sim	9.46	11.14	15.31	19.8
RPMI8226	Ven + Sim	0.95	3.15	2.39	6.58
NCIH929	Ven + Sim	0.5	1.7	3.75	6.78
U266	Ven + Sim	0.75	2.07	1.57	2.82
MOLP8	Ven + Sim	-5.9	-3.69	1.13	2.69
NCIH929	S63845 + Sim	8.89	13.27	15.52	24.83
OPM2	S63845 + Sim	8.24	14.05	11.62	20
L363	S63845 + Sim	8.58	13.65	10.98	17.24
MOLP8	S63845 + Sim	3.14	5.88	10.4	15.86
RPMI8226	S63845 + Sim	3.89	6.25	8.5	12.95
KMS12PE	S63845 + Sim	1.66	4.28	5.02	10.66
U266	S63845 + Sim	0.67	2.44	1.79	3.84
