# Supplementary material for: Statin-induced Mitochondrial Priming Sensitizes Multiple Myeloma Cells to BCL2 and MCL-1 Inhibitors
Source: Cancer Res Commun. 2023 Dec 8;3(12):2497–509. doi: 10.1158/2767-9764.CRC-23-0350 (PMC10704957; doi:10.1158/2767-9764.CRC-23-0350)
Supplement: Table S9 — Supplementary Table S9 shows the cytogenetic characteristics of the MM patients whose bone marrow cells were studied in Figure 4D. [file crc-23-0350-s22.pdf]

Table S9: MM patient cell cytogenetic abnormalities of ex vivo treated patient samples. Order corresponds with Figure 4D. Top six patient samples were sensitized to venetoclax treatment by statins whereas the bottom six were not.

**Patient**	**Cytogenetics**
2263	t(11;14), +1, -13, del(17p)
2269	+1q, -13
1350	t(11;14)
1466	+3, +7, +9, del13q, del(17p)
1198	+1q, -13
1869	t(4;14)
1724	vt(11;14), +1p, +1q, +3, +7, +9, -13, del(17p)
1810	+7, +9, +11, del(13q), +14q
1890	t(4;14), +1q, +3, +9, +11, -13
1865	+1p, +1q, +3, +7, +9, +11, del(17p), +17
1880	+1q, +3,+7,+9,-13,+14q
1808	+1q, +3,+7,+9,+11,+14q, del(17p)
